# Mapping cellular cytosolic pH *in vivo* under physiological and pathophysiological conditions

**DOI:** 10.1016/j.jbc.2025.110637

**Published:** 2025-08-28

**Authors:** Shuwei Zhang, Xiaoxin Hu, Bowen Zhang, Jingxuan Liu, Hexi Feng, Caiying Liu, Yi Hui, Yujiang Fang, Ling Liu, Xiaoqing Zhang

**Affiliations:** 1 Translational Research Institute of Brain and Brain-Like Intelligence, Shanghai Fourth People's Hospital, School of Medicine, Tongji University, Shanghai, China; 2 Translational Medical Center for Stem Cell Therapy, Shanghai East Hospital, School of Medicine, Tongji University, Shanghai, China; 3 Shanghai Institute of Stem Cell Research and Clinical Translation, School of Medicine, Tongji University, Shanghai, China; 4 Key Laboratory of Neuroregeneration of Shanghai Universities, School of Medicine, Tongji University, Shanghai, China; 5 Stem Cell Research Center, School of Medicine, Tongji University, Shanghai, China; 6 Key Laboratory of Spine and Spinal Cord Injury Repair and Regeneration of Ministry of Education, School of Medicine, Tongji University, Shanghai, China; 7 Clinical Center for Brain and Spinal Cord Research, Tongji University, Shanghai, China

**Keywords:** Smad5, nucleocytoplasmic distribution, cytosolic pH, physiology, pathophysiology

## Abstract

Cytosolic pH (pHc) is one of the most essential intracellular microenvironments profoundly affecting charges, conformations and therefore functions of macromolecules. It remains challenging to quantitatively measure pHc at single-cell resolution *in vivo*, and how pHc of a specific cell type dynamically responds to physiological or pathophysiological fluctuations remains elusive. Smad5 is a pHc sensor with accelerated nuclear export upon pHc alkalization and ideally the nucleocytoplasmic ratio of Smad5 would be a potential indicator of pHc. Here, we revealed that the constitutive eGFP-Smad5 expression transgenic mice could serve as pHc reporter for quantitative detection of cellular pHc *in vivo*. We mapped pHc of major cell types in the reporter mice and found robust pHc heterogeneity in individual cell types or different cell types adjacently located within a tissue. By acute systemic acidification or alkalization challenges, we also characterized pHc-stable and pHc-sensitive cell types. In pathological microenvironments related to type 2 diabetes mellitus or tumors, pHc showed responsive or adaptive changes in some specific cell types, highlighting pathophysiological roles of pHc in disease onset and progression or plasticity of cells under chronic disease states. Our study establishes a previously unachievable method for mapping of cellular pHc *in vivo* under both physiological and pathophysiological conditions.

Virtually, the charge and conformation of all macromolecules harboring acidic or basic moieties are influenced by surrounding pH (1, 2). Meanwhile, protons (H^+^) partake in numerous metabolic reactions and serve as a source of electrochemical energy in driving the transmembrane transport of many organic and inorganic substrates (3, 4). Extracellular pH (pHe) and cytosolic pH (pHc), therefore, exert essential roles in biochemical reactions as well as cellular functions, and they must be either maintained or purposely regulated in specific biological contexts (5, 6, 7).

Most chemical reactions happen inside the cell or at the cell membrane, and as compared with pHe, pHc is even more dynamically regulated and tightly correlated with biological functions of a cell (3). Clinical observations reveal that patients with an abnormal pH in arterial blood gases reach about 40% (8). In diseases like sepsis, chronic pancreatitis, cancer, diabetic ketoacidosis, and vaginitis, the pH levels in blood or tissue fluid deviate from normal ranges (9, 10, 11, 12, 13). Thus, cells would have evolved to adopt mechanisms to timely respond or adapt to environmental pH oscillations, and abnormal pHc caused by misregulation of the cell would play a role in disease onset and progression.

Monitoring pHc in specific cell types under basal conditions, physiological activities, or disease states is the first step to study pHc physiology and pathophysiology. Several methods have been developed for monitoring pHc in cells (14, 15, 16, 17, 18, 19, 20). Inserting a microelectrode into a cell is used for pHc detecting, while only applicable for cells with a large body size in specific species (14, 17). Moreover, puncture during microelectrode insertion always mixes intracellular fluid with extracellular fluid, likely resulting in inaccurate pHc measurement. Noninvasive pHc imaging can be achieved by using magnetic resonance imaging (MRI). However, MRI is only feasible to reflect the pHc of a homogenous population of cells in a defined tissue area, but not the pHc of an individual cell, and the detected pHc value could be influenced by variable compounds distributed in the interstitial fluid (16, 18, 19, 21, 22). Fluorescence spectroscopy is thus far the only available approach to measure pHc at single-cell level; however, this method could only be applied in cultured cells. While pH-sensitive green fluorescent protein can be expressed in animals, the standard curve of fluorescence intensity and pH cannot be established *in vivo*. Monitoring pHc in a quantitative way in different tissues at the single-cell level *in vivo* still remains an urgent need (23, 24, 25, 26).

We have reported that Smad5 is a pHc sensor, which can actively respond to pHc changes through nucleocytoplasmic shuttling. Two acidic amino acid clusters and one basic amino acid cluster in the MH1 domain of Smad5 are responsible for this pHc sensing. Increasing in pHc leads to immediate nuclear export of Smad5, while decreasing in pHc slows down its nuclear export and ends up with Smad5 rapid nuclear accumulation (27, 28, 29). These suggest that states in nucleocytoplasmic distribution of Smad5 may be used to report cellular pHc both in *in vitro* cultured cells and more importantly *in vivo.*

Here, we validated that the ratio of Smad5 nucleocytoplasmic distribution is a general indicator of pHc in different cell types. We constructed a mouse model to report pHc in all types of somatic cells *in vivo* by integrating an eGFP-Smad5 expression cassette at the mouse Rosa26 locus. Based on the nucleocytoplasmic ratio of eGFP-Smad5, we quantified pHc in major cell types in mice under basal conditions, systemic acidification or alkalization conditions, and type 2 diabetes mellitus (T2DM), together with pHc in infiltrated immune cells in xenografted tumor tissues. Our study establishes a previously unachievable method for pHc measurement at single-cell resolution and draws a pHc atlas of major cell types in mice. We also present evidence and show that several cell types actively or passively change their pHc upon physiological or pathophysiological conditions, implying important biological functions of pHc in health and disease.

## Results

### Nucleocytoplasmic ratio of eGFP-Smad5 ectopically expressed in cultured cells is a valid indicator of pHc

**Figure 1:**
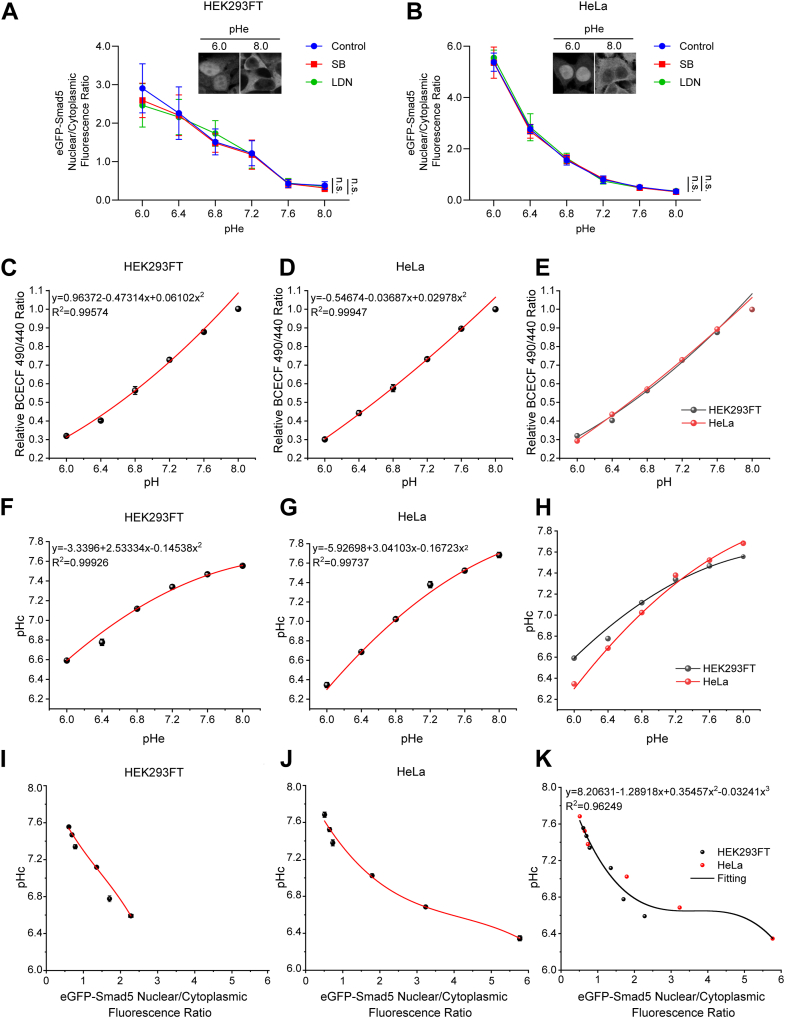
**Establishing of the mathematic model between eGFP-Smad5 nucleocytoplasmic ratio and pHc**. *A and B*, eGFP-Smad5 nucleocytoplasmic fluorescence ratio quantification at various pHe in HEK293FT cells (*A*) and HeLa cells (*B*) of control, SB431542 pretreatment, and LDN193189 pretreatment groups. Data are presented as mean ± SD; statistical analysis was performed using one-way ANOVA and no significant difference was observed; n = 20 cells for all groups. *C and D*, standard curves of fluorescence intensity ratio of BCECF *versus* the buffered pH in HEK293FT cells (*C*) and HeLa cells (*D*) permeabilized with Nigericin to equilibrate pHe and pHc. Data are presented as mean ± SD; n = 3 independent experiments. *E*, HEK293FT cells (*black circles*) and HeLa cells (*red circles*) show identical correlations between fluorescence intensity ratio of BCECF *versus* equilibrated cytosolic pH after Nigericin permeabilization. *F and G*, calibrated pHc according to the fluorescence intensity ratio of BCECF in HEK293FT cells (*F*) and HeLa cells (*G*) at different pHe in the absence of Nigericin permeabilization. Data are presented as mean ± SD; n = 3 independent experiments. *H*, intact HEK293FT cells (*black circles*) and HeLa cells (*red circles*) show variable pHc responsiveness to pHe challenges. *I and J*, standard curves of pHc *versus* eGFP-Smad5 nucleocytoplasmic ratio in HEK293FT cells (*I*) and HeLa cells (*J*). Data are presented as mean ± SD; n = 3 independent experiments. *K*, the mathematic model of pHc *versus* eGFP-Smad5 nucleocytoplasmic ratio was fitted from HEK293FT cells and HeLa cells. According to regression analysis, the mathematic model was established as: y = 8.20631 − 1.28918x + 0.35457x^2^ − 0.03241x^3^, where y represents pHc and x represents nucleocytoplasmic ratio of eGFP-Smad5. R^2^ = 0.96249.

As a pHc sensor, Smad5 responds to pHc changes through nucleocytoplasmic shuttling. Meanwhile, Smad5 is one of the downstream signaling molecules of the transforming growth factor-β (TGFβ) superfamily (30, 31). Immortalized human embryonic kidney cells (HEK293FT) and cervical cancer (HeLa) cells stably expressing eGFP-Smad5 were cultured in medium with variable adjusted pHe (pH 6.0, pH 6.4, pH 6.8, pH 7.2, pH 7.6, and pH 8.0). We found that eGFP-Smad5 was mostly located in the nucleus when pHe was 6.0, whereas with a pHe higher than 7.6, it was mostly distributed in the cytosol (Fig. 1, *A* and *B*). Similar results were observed *via* Western blot analysis following fractionation of cytoplasmic and nuclear proteins from HEK293FT cells and HeLa cells exposed to varying pHe (Fig. S1, *A–D*). Pretreatment with SB431542 (SB) or LDN193189 (LDN), selective inhibitors of TGFβ superfamily type I activin receptor-like kinase receptors and bone morphogenetic protein (BMP) type I receptors, had no effect on Smad5 nucleocytoplasmic distribution at different buffering pHe (Fig. 1, *A* and *B*). These data suggest that nucleocytoplasmic distribution of Smad5 is tied to environmental pH and is largely independent of TGFβ signaling and BMP signaling.

We next asked whether the nucleocytoplasmic ratio of eGFP-Smad5 could serve as a valid and stable indicator for pHc. To this end, eGFP-Smad5 expressed HEK293FT cells and HeLa cells were incubated with BCECF, a cell-permeant and dual-excitation ratiometric pH indicator. Cells were then incubated in buffers in a wide range of pH (pH 6.0, pH 6.4, pH 6.8, pH 7.2, pH 7.6, and pH 8.0) with protonophore Nigericin to equilibrate the pHc to the pHe of the buffer (24, 32). As expected, BCECF fluorescence ratio (R_490/440_) linearly correlated with pHc and a standard curve of pHc to BCECF fluorescence ratio was subsequently generated in HEK293FT cells and HeLa cells (Fig. 1, *C* and *D*). In addition, HEK293FT cells and HeLa cells showed almost exactly the same standard curve of pHc to BCECF fluorescence ratio, suggesting the robustness and a general capacity of BCECF to accurately indicate pHc in different cell types (Fig. 1*E*).

Based on the standard curve generated, we studied pHc by calculating BCECF fluorescence ratio in both HEK293FT cells and HeLa cells under variable pHe buffering conditions in the absence of the protonophore Nigericin. In both cell types, pHc increased or decreased in the same direction as of the pHe changes (Fig. 1, *F*–*H*). We then quantified the nucleocytoplasmic ratio of eGFP-Smad5 under different pHe exposure conditions and the functions between the nucleocytoplasmic ratio of eGFP-Smad5 and pHc were calculated in both HEK293FT cells and HeLa cells (Fig. 1, *I* and *J* and Fig. S1, *E* and *F*). A well-fitting mathematical model could be derived as: y = 8.20631 − 1.28918x + 0.35457x^2^ − 0.03241x^3^ in both cell types, where y represents pHc and x represents nucleocytoplasmic ratio of eGFP-Smad5 (Fig. 1*K*). These data suggest that by measuring the nucleocytoplasmic ratio of eGFP-Smad5, pHc could be calculated in a quantitative manner at single cell level. We also observed that as a tumor cell line, HeLa cells exhibited greater pHc fluctuations in response to both acidic and basic pHe challenges compared to HEK293FT cells, suggesting distinct pHc adaptation capacities between these two cell types (Fig. 1*H*). Taken together, we established a feasible system to map pHc at single cell level by monitoring the nucleocytoplasmic ratio of eGFP-Smad5.

### Constructing eGFP-Smad5 transgenic mice for *in vivo* pHc measurement

**Figure 2:**
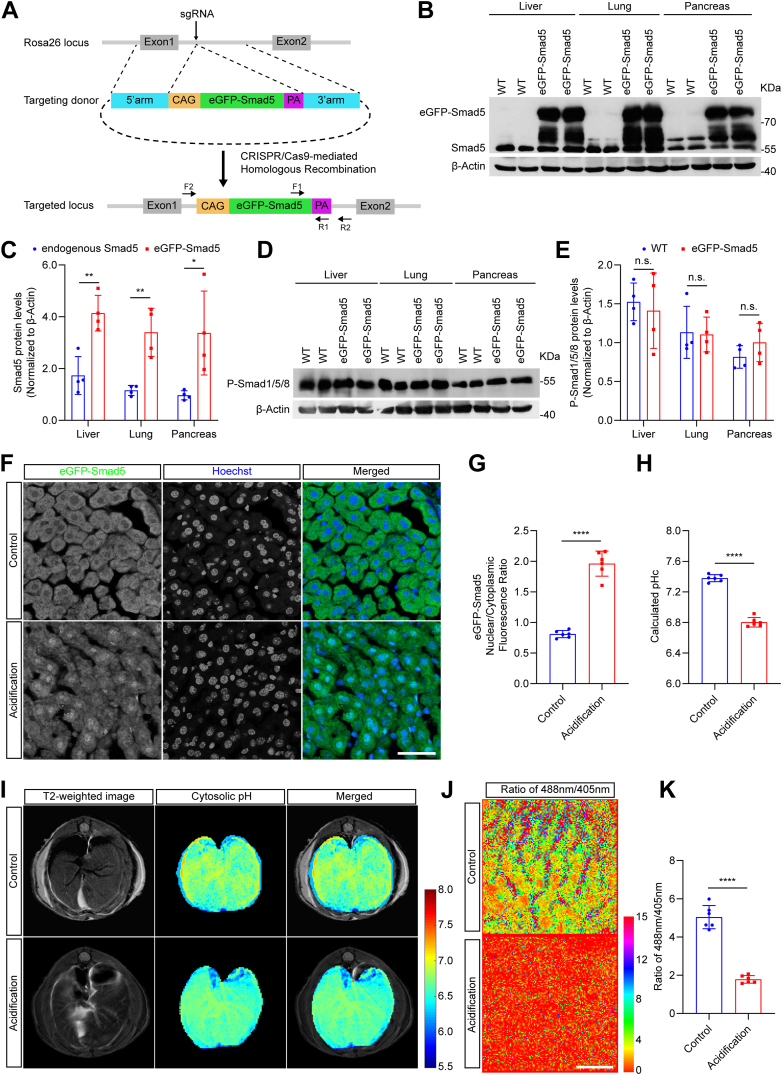
**Generation of pHc reporter mice**. *A*, schematic overview of CRISPR/Cas9-mediated knock-in of the CAG-eGFP-Smad5 cassette at the Rosa26 locus. The *top* panel shows the organization of mice Rosa26 genomic locus. The *middle panel* shows the design of the Rosa26-CAG-eGFP-Smad5 targeting donor vector. The *bottom panel* shows the design of CRISPR/Cas9-mediated genomic targeting and homologous recombination. *B*, Western blot analysis of endogenous Smad5 and ectopically expressed eGFP-Smad5 in WT and pHc reporter mice. *C*, endogenous Smad5 and eGFP-Smad5 protein expression were quantitated by densitometry. β-Actin protein expression was used to normalize. Data are presented as mean ± SD; ∗*p* < 0.05, ∗∗*p* < 0.01, unpaired two-tailed *t* test; n = 4 for both groups. *D*, Western blot analysis of phosphorylated endogenous Smad1/5/8 in tissues from WT and pHc reporter mice. *E*, P-Smad1/5/8 protein expression were quantitated by densitometry. β-Actin protein expression was used to normalize. Data are presented as mean ± SD; n.s., no significant difference, unpaired two-tailed *t* test; n = 4 for both groups. *F*, representative immunofluorescence staining of eGFP-Smad5 (*green*) and Hoechst 33,258 (*blue*) in liver under control and systemic acidification conditions of pHc reporter mice. Scale bar, 50 μm. *G*, eGFP-Smad5 nucleocytoplasmic fluorescence ratio quantification in (*G*). Data are presented as mean ± SD; ∗∗∗∗*p* < 0.0001, unpaired two-tailed *t* test; n = 6 for both groups. *H*, pHc calculation based on eGFP-Smad5 nucleocytoplasmic ratio in (*G*). Data are presented as mean ± SD; ∗∗∗∗*p* < 0.0001, unpaired two-tailed *t* test; n = 6 for both groups. *I*, representative magnetic resonance images and pH map in the liver of mice under control and systemic acidification conditions. *J*, liver BCECF fluorescence intensity ratio (488 nm/405 nm) under control and systemic acidification conditions. Scale bar, 12.5 μm. *K*, quantification of liver BCECF fluorescence intensity ratio in (*J*). Data are presented as mean ± SD; ∗∗∗∗*p* < 0.0001, unpaired two-tailed *t* test; n = 6 for both groups.

To study pHc in different cell types *in vivo* under either healthy or diseased conditions, we constructed eGFP-Smad5 transgenic mice. The Rosa26-CAG-eGFP-Smad5 donor plasmid, in which the CAG-eGFP-Smad5 expression cassette was flanked by 5′- and 3′-Rosa26 homologous arms, was used for CRISPR/Cas9-mediated homologous recombination (HR). A sgRNA (GCAGGCTTAAAGGCTAACC) was used to target the intron one within the Rosa26 locus. Cas9 expression vector, Rosa26 targeting sgRNA and Rosa26-CAG-eGFP-Smad5 donor plasmid were co-microinjected into mice zygotes, which were subsequently transferred to pseudopregnant females (Fig. 2*A*). F1 founders were validated by genomic DNA PCR and were further used to generate heterozygous or homozygous eGFP-Smad5 transgenic mice (Fig. S2*A*). Western blot analysis revealed that in transgenic mice, eGFP-Smad5 was expressed in all types of tissues analyzed (Fig. S2*B*), and the expression levels of overexpressed eGFP-Smad5 were 2 to 4 folds higher than those of endogenous Smad5 (Fig. 2, *B* and *C*). In addition, overexpression of eGFP-Smad5 had no effect on the phosphorylation pattern of endogenous regulatory Smads (p-Smad1/5/8) in transgenic mice (Fig. 2, *D* and *E*). Body weight gain and food uptake in transgenic mice and WT littermates were comparable (Fig. S2, *C* and *D*), and they showed similar insulin sensitivity as determined by insulin tolerance test (ITT) (Fig. S2, *E* and *F*). eGFP-Smad5 transgenic mice showed slightly improved glucose tolerance as determined by glucose tolerance test (GTT) (Fig. S2, *G* and *H*), likely due to a better capacity in insulin processing and secretion as previous described (33). Rotarod test revealed that transgenic mice and WT mice exhibited similar capacities in motor coordination and balance (Fig. S2*I*). Serum biochemistry studies also showed that transgenic mice had normal liver, heart and kidney functions (Fig. S2, *J*–*L*). Together, these data indicate that the constructed transgenic mice are phenotypically normal and could serve as reporter mice for *in vivo* pHc mapping.

Reporter mice were immediately cardiac perfused with 37 °C prewarmed 4% paraformaldehyde (PFA) after anesthesia. According to the nucleocytoplasmic ratios of eGFP-Smad5, the calculated pHc in spleen, liver and muscle cells highly coincided with their homogenate pH (Fig. S3, *A*–*C*). The transgenic mice were then intraperitoneally injected with lactic acid to induce systemic acidification. We observed robust eGFP-Smad5 nuclear accumulation in liver cells and the putative pHc according to the functions established above dropped from 7.38 to 6.80 (Fig. 2, *F*–*H*). Amine and amide concentration-independent detection (AACID) with MRI has been used to measure pHc of liver tissue *in vivo* although this method could not detect pHc at single-cell resolution (22, 34, 35). MRI results showed that the collective pHc of liver tissue in control and acidification groups dropped from 7.23 to 6.55 (Fig. 2*I*), which is quite close to above results obtained from reporter mice. We also tail vein injected BCECF to mice and observed a decrease of the ratio of 488 nm/405 nm after lactic acid-induced systemic acidification, indicating decreased pHc in liver cells (Fig. 2, *J* and *K*). However, it is not possible to obtain a pH standard curve of the BCECF probe *in vivo*, thereby making it impossible to precisely calculate pHc in liver cells. These data suggest that the constructed eGFP-Smad5 transgenic mice enable unprecedented measurement of pHc at single cell resolution *in vivo*.

### Mapping pHc in major cell types in reporter mice under baseline conditions

To study pHc of major cell types, three male reporter mice with 6 to 8 weeks of age were immediately cardiac perfused with 37 °C PFA after anesthesia. Cell types in different tissues were immunolabeled with antibodies targeting GFP and featured proteins, and nuclei were counterstained with Hoechst 33,258. In most cases, β-catenin was labeled to mark the membrane boundary of a cell. Nucleocytoplasmic ratios of eGFP-Smad5 and pHc were therefore measured in individual cell types.

**Figure 3:**
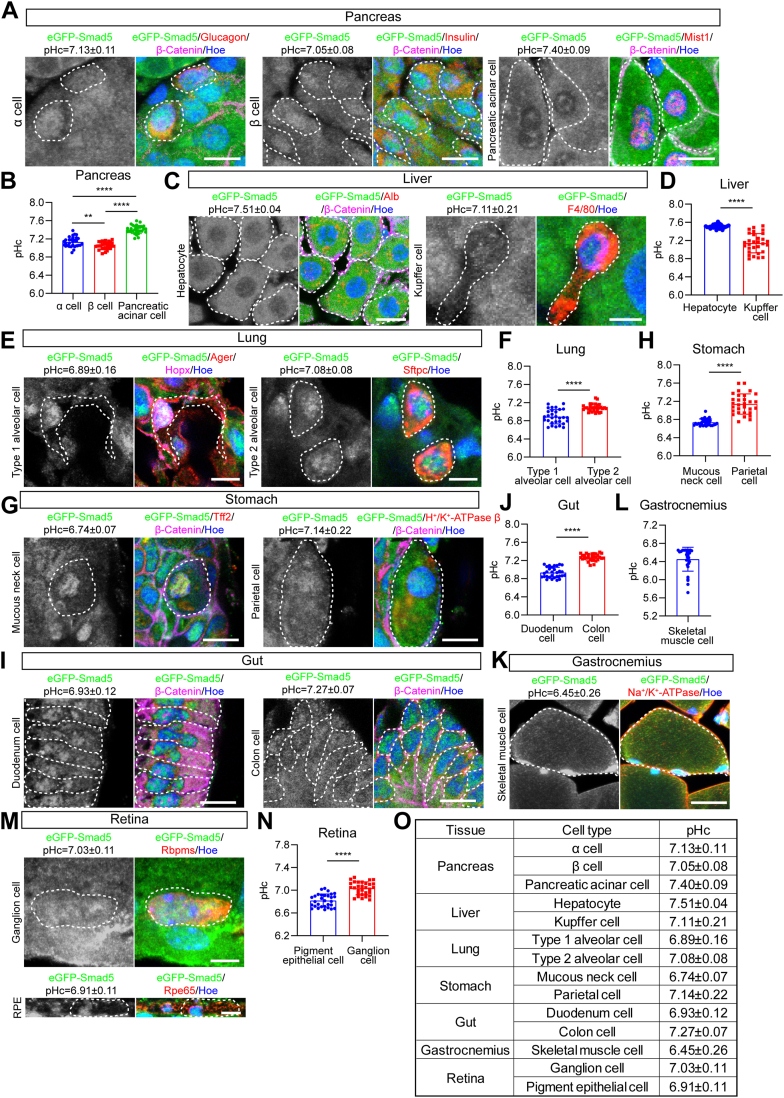
**Mapping pHc of major cell types in pHc reporter mice.***A*, representative immunofluorescence staining of eGFP-Smad5 (*green*), cell type specific markers (Glucagon for α cell; Insulin for β cell; Mist1 for pancreatic acinar cell, *red*), β-catenin (labeling plasma membrane boundary, *magenta*), and Hoechst 33,258 (*blue*) in pancreatic tissues of pHc reporter mice. Scale bars, 10 μm. *B*, pHc calculation based on eGFP-Smad5 nucleocytoplasmic ratio in (*A*). Data are presented as mean ± SD; ∗∗*p* < 0.01, ∗∗∗∗*p* < 0.0001, unpaired two-tailed *t* test; n = 30 cells for all groups. *C*, representative immunofluorescence staining of eGFP-Smad5 (*green*), cell type specific markers (Alb for hepatocyte; F4/80 for Kupffer cell, *red*), β-catenin (*magenta*), and Hoechst 33,258 (*blue*) in liver tissues of pHc reporter mice. Scale bars, 12.5 μm for the *left panel* and 4 μm for the *right panel*. *D*, pHc calculation based on eGFP-Smad5 nucleocytoplasmic ratio in (*C*). Data are presented as mean ± SD; ∗∗∗∗*p* < 0.0001, unpaired two-tailed *t* test; n = 30 cells for both groups. *E*, representative immunofluorescence staining of eGFP-Smad5 (*green*), cell type specific markers (Hopx and Ager for type 1 alveolar cell, *red* and *magenta*, respectively; Sftpc for type 2 alveolar cell, *red*), and Hoechst 33,258 (*blue*) in lung tissues of pHc reporter mice. Scale bars, 8 μm for the *left panel* and 6 μm for the *right panel*. *F*, pHc calculation based on eGFP-Smad5 nucleocytoplasmic ratio in (*E*). Data are presented as mean ± SD; ∗∗∗∗*p* < 0.0001, unpaired two-tailed *t* test; n = 30 cells for both groups. *G*, representative immunofluorescence staining of eGFP-Smad5 (*green*), cell type specific markers (Tff2 for mucous neck cell; H^+^/K^+^-ATPase β for parietal cell, *red*), β-catenin (*magenta*), and Hoechst 33,258 (*blue*) in stomach tissues of pHc reporter mice. Scale bars, 10 μm. *H*, pHc calculation based on eGFP-Smad5 nucleocytoplasmic ratio in (*G*). Data are presented as mean ± SD; ∗∗∗∗*p* < 0.0001, unpaired two-tailed *t* test; n = 30 cells for both groups. *I*, representative immunofluorescence staining of eGFP-Smad5 (*green*), β-catenin (*magenta*), and Hoechst 33,258 (*blue*) in gut tissues of pHc reporter mice. Scale bars, 10 μm. *J*, pHc calculation based on eGFP-Smad5 nucleocytoplasmic ratio in (*I*). Data are presented as mean ± SD; ∗∗∗∗*p* < 0.0001, unpaired two-tailed *t* test; n = 30 cells for both groups. *K*, representative immunofluorescence staining of eGFP-Smad5 (*green*), cell type specific marker (Na^+^/K^+^-ATPase, *red*), and Hoechst 33,258 (*blue*) in gastrocnemius tissues of pHc reporter mice. Scale bar, 20 μm. *L*, pHc calculation based on eGFP-Smad5 nucleocytoplasmic ratio in (*K*). Data are presented as mean ± SD; n = 30 cells. *M*, representative immunofluorescence staining of eGFP-Smad5 (*green*), cell type specific markers (Rbpms for ganglion cell; Rpe65 for pigment epithelial cell, *red*), and Hoechst 33,258 (*blue*) in retinal tissues of pHc reporter mice. Scale bars, 6 μm for the *top* panel and 10 μm for the *bottom panel*. *N*, pHc calculation based on eGFP-Smad5 nucleocytoplasmic ratio in (*M*). Data are presented as mean ± SD; ∗∗∗∗*p* < 0.0001, unpaired two-tailed *t* test; n = 30 cells for both groups. *O*, summary table of baseline pHc of 14 major cell types analyzed.

We measured pHc in 14 major cell types of eight tissues in total and obtained pHc with a range of 6.45 to 7.51 in all the cell types tested (Fig. 3, *A*–*O*), *i.e.* in the pancreas, glucagon^+^ α cells: 7.13 ± 0.11, insulin^+^ β cells: 7.05 ± 0.08, Mist1^+^ acinar cells: 7.40 ± 0.09; in the liver, Alb^+^ hepatocytes: 7.51 ± 0.04, F4/80^+^ Kupffer cells (macrophage): 7.11 ± 0.21; in the lung, Ager^+^/Hopx^+^ type 1 alveolar cells: 6.89 ± 0.16, Sftpc^+^ type 2 alveolar cells: 7.08 ± 0.08; in the stomach, Tff2^+^ mucous neck cells: 6.74 ± 0.07, H^+^/K^+^-ATPase β^+^ parietal cells: 7.14 ± 0.22; in the duodenum cells: 6.93 ± 0.12; in the colon cells: 7.27 ± 0.07; in the Na^+^/K^+^-ATPase^+^ gastrocnemius muscle cells: 6.45 ± 0.26; in the retina, Rbpms^+^ ganglion cells: 7.03 ± 0.11, Rpe65^+^ retina pigment epithelial (RPE) cells: 6.91 ± 0.11 (Fig. 3, *A*–*O* and Fig. S4–S10).

Baseline pHc of each cell type was consistent in the three mice tested. In the same tissue, different cell types showed phenotype-specific characteristics even they were distributed in the same local area (Fig. 3, *B*, *D*, *F*, *H*, *J* and *N*). This suggests that each cell type has their own machinery in adjusting pHc to maintain their normal function. Skeletal muscle cells had the lowest pHc, likely due to their active activity and specific metabolic states (36). In the stomach, parietal cells are responsible for gastric acid secretion, which aids in the digestion of food, absorption of minerals, and control of harmful bacteria (37). On the other hand, mucous neck cells secrete alkaline mucus to protect against the secreted acid and pepsin by forming a continuous layer of gastric mucus (38). These not only explain the acidic pHc in mucous neck cells and the alkalized pHc in parietal cells but also strengthen the accuracy of the method we developed for detecting pHc *via* measuring the nucleocytoplasmic ratios of eGFP-Smad5. Meanwhile, although most cell types showed homogeneity in pHc levels, Kupffer cells in the liver, type 1 alveolar cells in the lung, parietal cells in the stomach, and cells in the gastrocnemius muscle showed large deviations of their pHc, highlighting their heterogeneous functional states.

### Characterization of pHc-stable and pHc-sensitive cell types upon acute systemic acidification and alkalization challenges

**Figure 4:**
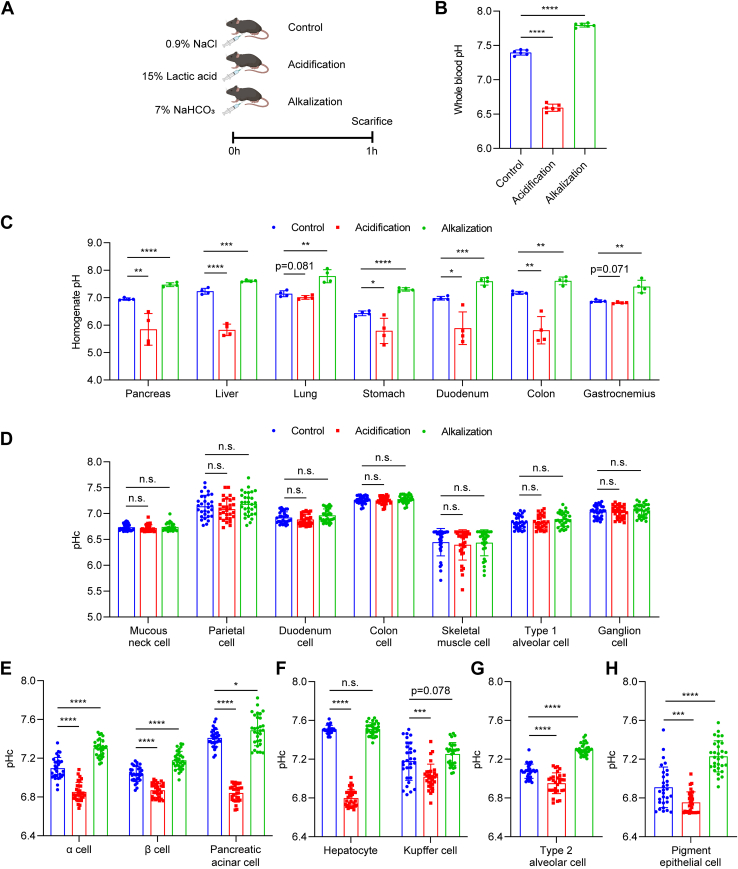
**pHc changes upon acute systemic acidification and alkalization challenges**. *A*, schematic overview of constructing mouse models of acute systemic acidification and alkalization. *B*, whole blood pH in mice of control and systemic acidification or alkalization challenged groups. Data are presented as mean ± SD; ∗∗∗∗*p* < 0.0001, unpaired two-tailed *t* test; n = 6 for all groups. *C*, homogenate pH of tissues of control, acute systemic acidification or alkalization challenged groups. Data are presented as mean ± SD; n.s., no significant difference, ∗*p* < 0.05, ∗∗*p* < 0.01, ∗∗∗*p* < 0.001, ∗∗∗∗*p* < 0.0001, unpaired two-tailed *t* test; n = 4 for all groups. *D*, pHc-stable cell types show steady pHc under either systemic acidification or systemic alkalization conditions. Data are presented as mean ± SD; n.s., no significant difference, unpaired two-tailed *t* test; n = 30 cells for all groups. *E–H*, pHc-sensitive cell types show pHc changes at the same direction under either systemic acidification or systemic alkalization condition. Data are presented as mean ± SD; n.s., no significant difference, ∗*p* < 0.05, ∗∗∗*p* < 0.001, ∗∗∗∗*p* < 0.0001, unpaired two-tailed *t* test; n = 30 cells for all groups.

The normal blood pH is between 7.35 and 7.45, and acidemia and alkalemia are pathophysiological states that appeared in many severe acute or chronic diseases (39, 40). We recapitulated systemic acidification and alkalization states by acute intraperitoneal injection of lactic acid and sodium bicarbonate in pHc reporter mice (Fig. 4, *A* and *B*). The homogenate pH of tissues changed in the same direction as of the systemic pH changes (Fig. 4*C*). Mucous neck cells, parietal cells, duodenum cells, colon cells, skeletal muscle cells, type 1 alveolar cells, and ganglion cells remained stable pHc under both systemic acidification and alkalization conditions (Fig. 4*D*). Meanwhile, cells in the pancreas (Fig. 4*E* and Fig. S11) and liver (Fig. 4*F* and Fig. S12), type 2 alveolar cells (Fig. 4*G* and Fig. S13) in the lung, and RPE cells (Fig. 4*H* and Fig. S14) in the retina showed pHc changes in the same direction as of the systemic pH changes. These data suggest that the pHc-stable cells are resistant to environmental pH changes, while pHc-sensitive cell types are ready to sense and therefore respond to these environmental pH fluctuations.

### Cell type-specific pHc changes in type 2 diabetes mellitus

**Figure 5:**
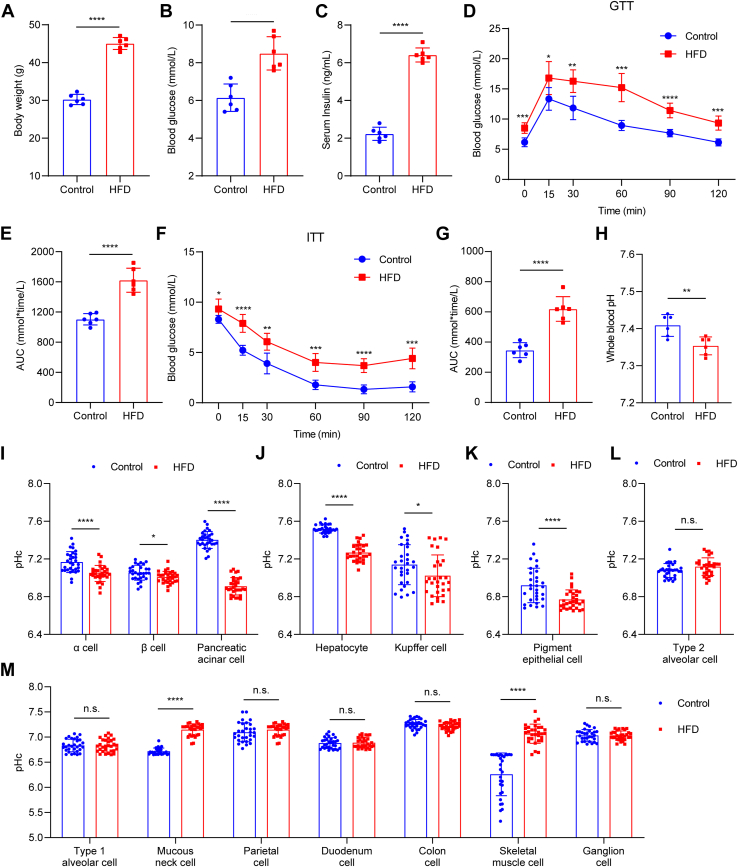
**Aberrant pHc in pHc-sensitive cell types in type 2 diabetes mellitus**. *A*, body weight in control pHc reporter mice and HFD-treated pHc reporter mice. Data are presented as mean ± SD; ∗∗∗∗*p* < 0.0001, unpaired two-tailed *t* test; n = 6 for both groups. *B*, fasting blood glucose levels in control and HFD pHc reporter mice. Data are presented as mean ± SD; ∗∗∗*p* < 0.001, unpaired two-tailed *t* test; n = 6 for both groups. *C*, serum insulin levels in control and HFD pHc reporter mice. Data are presented as mean ± SD; ∗∗∗∗*p* < 0.0001, unpaired two-tailed *t* test; n = 6 for both groups. *D*, glucose tolerance test in control and HFD pHc reporter mice. Data are presented as mean ± SD; ∗*p* < 0.05, ∗∗*p* < 0.01, ∗∗∗*p* < 0.001, ∗∗∗∗*p* < 0.0001, unpaired two-tailed *t* test; n = 6 for both groups. *E*, area under curve (AUC) in (*D*). Data are presented as mean ± SD; ∗∗∗∗*p* < 0.0001, unpaired two-tailed *t* test; n = 6 for both groups. *F*, insulin tolerance test in control and HFD pHc reporter mice. Data are presented as mean ± SD; ∗*p* < 0.05, ∗∗*p* < 0.01, ∗∗∗*p* < 0.001, ∗∗∗∗*p* < 0.0001, unpaired two-tailed *t* test; n = 6 for both groups. *G*, AUC in (*F*). Data are presented as mean ± SD; ∗∗∗∗*p* < 0.0001, unpaired two-tailed *t* test; n = 6 for both groups. *H*, whole blood pH in control and HFD-treated pHc reporter mice. Data are presented as mean ± SD; ∗∗*p* < 0.01, unpaired two-tailed *t* test; n = 6 for both groups. *I*, pHc changes in pancreatic α cell, β cell and acinar cell in control and HFD pHc reporter mice. Data are presented as mean ± SD; ∗*p* < 0.05, ∗∗∗∗*p* < 0.0001, unpaired two-tailed *t* test; n = 30 cells for both groups. *J*, pHc changes in hepatocyte and Kupffer cell in the liver in control and HFD pHc reporter mice. Data are presented as mean ± SD; ∗*p* < 0.05, ∗∗∗∗*p* < 0.0001, unpaired two-tailed *t* test; n = 30 cells for both groups. *K*, pHc changes in pigment epithelial cell in the retina in control and HFD pHc reporter mice. Data are presented as mean ± SD; ∗∗∗∗*p* < 0.0001, unpaired two-tailed *t* test; n = 30 cells for both groups. *L*, pHc changes in pulmonary type 2 alveolar cell in control and HFD pHc reporter mice. Data are presented as mean ± SD; n.s., no significant difference, unpaired two-tailed *t* test; n = 30 cells for both groups. *M*, pHc changes in pHc-stable cell types in control and HFD pHc reporter mice. Data are presented as mean ± SD; n.s., no significant difference, ∗∗∗∗*p* < 0.0001, unpaired two-tailed *t* test; n = 30 cells for both groups.

Obesity and type 2 diabetes mellitus (T2DM) belong to the most prevalent metabolic disorders, which always lead to long-lasting systemic acidification. To examine the pHc in pHc-sensitive cells in T2DM, reporter mice at 6 weeks of age were fed with high-fat diet (HFD) for 16 weeks (41, 42, 43). After HFD treatment, body weight gain (Fig. 5*A*), hyperglycemia (Fig. 5*B*), hyperinsulinemia (Fig. 5*C*), and abnormalities in GTT (Fig. 5, *D* and *E*) and ITT (Fig. 5, *F* and *G*) were observed. In addition, the whole blood pH in T2DM mice showed obvious acidification as compared with that in control mice (Fig. 5*H*).

pHc in most pHc-sensitive cell types, except for that in type 2 alveolar cells in the lung, showed an acidification phenotype in T2DM, coinciding with the acidified systemic pH (Fig. 5, *I*–*L* and Fig. S15–S18). These data indicate that pHc-sensitive cells also actively respond to disease-associated local microenvironments. These data also suggest that even under long-term adaptation in disease conditions, most pHc-sensitive cells do not evolve a mechanism to compensate for the misregulated microenvironment. The aberrantly presented pHc in liver cells, pancreatic cells, and RPE cells might be relevant to insulin resistance, insulin processing deficits, and age-related macular degeneration (AMD), pathologies frequently related to T2DM (44, 45, 46).

Although pHc-stable cells showed refractory pHc changes during acute systemic acidification or alkalization, mucous neck cells in the stomach and gastrocnemius muscle cells showed pHc alkalization in T2DM, deviating from the acidified systemic pH (Fig. 5*M* and Fig. S17–S21). These results highlight cell state and functional adaptation during the progression of T2DM, which is mirrored by the adaptive long-lasting pHc changes in these cells.

### Immune cells in the tumor microenvironment show cell type-specific pHc changes

**Figure 6:**
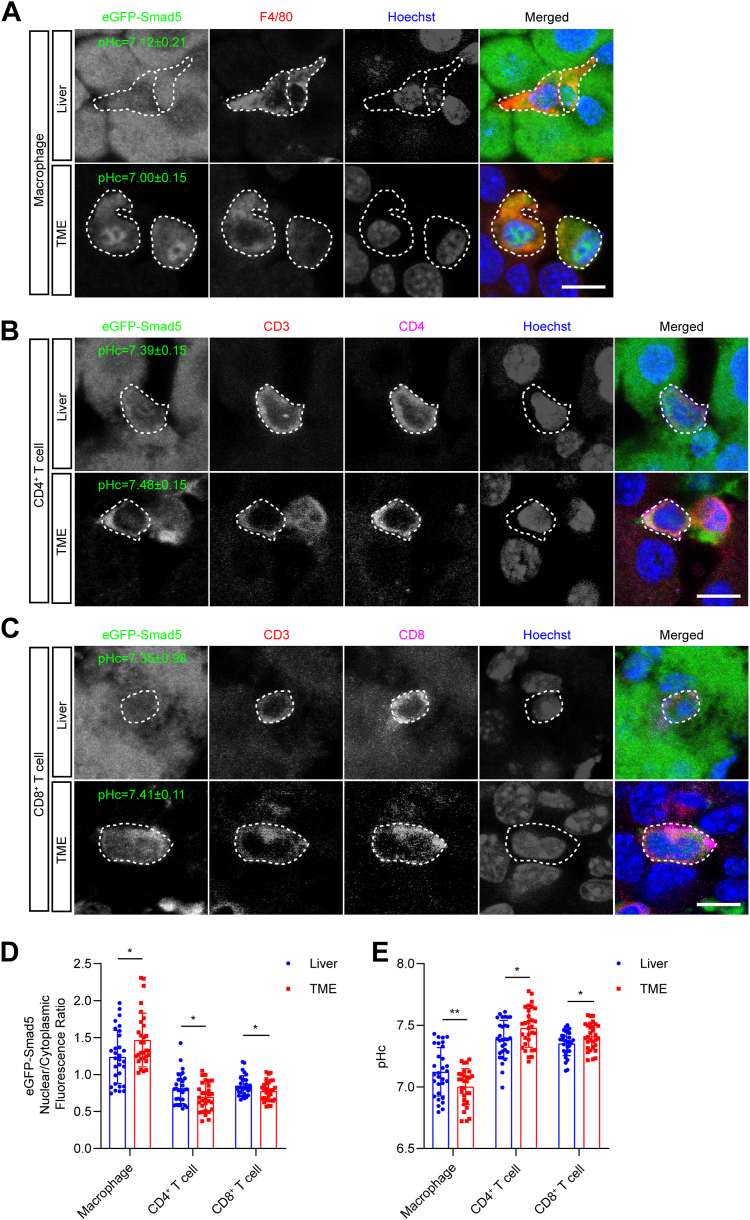
**pHc of immune cells in the tumor microenvironment.***A*, representative immunofluorescence staining of eGFP-Smad5 (*green*), F4/80 (*red*), and Hoechst 33,258 (*blue*) in macrophages in liver and TME. Scale bar, 10 μm. *B*, representative immunofluorescence staining of eGFP-Smad5 (*green*), CD3 (*red*), CD4 (*magenta*), and Hoechst 33,258 (*blue*) in CD4^+^ T cells in liver and TME. Scale bar, 10 μm. *C*, representative immunofluorescence staining of eGFP-Smad5 (*green*), CD3 (*red*), CD8 (*magenta*), and Hoechst 33,258 (*blue*) in CD8^+^ T cells in liver and TME. Scale bar, 10 μm. *D*, eGFP-Smad5 nucleocytoplasmic quantification of macrophages, CD4^+^ T cells, and CD8^+^ T cells in liver and TME. Data are presented as mean ± SD; ∗*p* < 0.05, unpaired two-tailed *t* test; n = 30 cells for all groups. *E*, pHc calculation based on eGFP-Smad5 nucleocytoplasmic ratio in (*D*). Data are presented as mean ± SD; ∗*p* < 0.05, ∗∗*p* < 0.01, unpaired two-tailed *t* test; n = 30 cells for all groups.

Tumor microenvironment (TME) contains a variety of infiltrating immune cells, such as macrophages and T cells, contributing substantially to tumor immunology (47, 48, 49). To quantify the pHc of immune cells in TME, we constructed a cell-line-derived xenograft (CDX) model of melanoma in eGFP-Smad5 transgenic mice using B16 melanoma cells (also C57BL/6 background). Infiltrated immune cells in the tumor mass were GFP^+^, while tumor cells were GFP^−^ (Fig. 6, *A*–*C*). We quantified pHc in three major cell types, *i.e.* F4/80^+^ macrophages: 7.00 ± 0.15, CD3^+^/CD4^+^ T cells: 7.48 ± 0.15, CD3^+^/CD8^+^ T cells: 7.41 ± 0.11 (Fig. 6, *A*–*E*). The pHc of macrophages in TME was lower than that of liver macrophages (Kupffer cells), whereas the pHc of CD3^+^/CD4^+^ T cells and CD3^+^/CD8^+^ T cells in TME were higher compared to liver local T cells. It is well-acknowledged that there is a prominent acidification in TME (12). The pHc acidification in TME macrophages coincides with their pHc-sensitive property. *In vitro* studies revealed that macrophages exhibited improved phagocytic function and enhanced IL-1β release under acidic culture conditions, and the lower pHc in TME macrophages might refer to an activated state (50, 51). Meanwhile, the alkalization of pHc in T cells *via* deletion of the anion exchanger 2 (AE2) or STS1 facilitated the activation of their anti-tumor immune functions (52, 53, 54). The higher pHc in infiltrated T cells suggests that T cells are not pHc-sensitive and the elevated pHc likely points to their activated state in the TME.

## Discussion

In this study, we established a previously unachievable method for the quantitative detection of pHc at single-cell resolution *in vivo* by detecting eGFP-Smad5 nucleocytoplasmic distribution in constructed pHc reporter mice. Based on this system, we have generated a detailed pHc atlas of major cell types in mice under baseline conditions. Although it has been considered that pHc is ∼7.2 in most normal cells (3, 4, 9, 12, 24), our study reveals that cells vary in their pHc within a wide range from 6.45 to 7.51. Cells harbor their own pHc nature, and there is a robust pHc heterogeneity in different cell types, even if they are adjacently placed. Integration of the pHc profiles at single-cell resolution into single-cell transcriptomics or single-cell epigenomics will therefore offer new insights into pHc biology, such as pHc regulatory mechanisms and biological implications of pHc in gene regulation or cell function (55, 56).

Based on acute systemic acidification or alkalization challenges, our study also reveals that cells have variable pHc sensing and responding sensitivity. Some cell types are resistant to environmental pH changes, and they maintain a stable pHc at either acidic or basic environmental pH. This indicates that maintaining pHc at an appropriate range might be crucial for executing normal physiological functions of these cells, such as mucous and acid secretion in neck cells or parietal cells, contraction of skeletal muscle cells, and electrical transmission in ganglion cells (37, 38, 57, 58). Different from these pHc-stable cell types, pHc-sensitive cell types respond to environmental pH changes in the same direction. pHc is usually regulated through ion channels on the cell membrane (3, 4). It is reasonable to hypothesize that pHc-stable and pHc-sensitive cells might harbor different pHc-regulatory ion channel profiles in order to timely balance or respond to environmental changes, although detailed mechanisms remain to be established.

pHc of α cells, β cells, and acinar cells in the pancreas, as well as that of hepatocytes and Kupffer cells in the liver, are prominently sensitive to acute environmental pH changes and systemic pH abnormalities caused by HFD-induced T2DM. The pHc fluctuations to systemic pH changes readily point to putative metabolic implications of pHc abnormality in these cell types. In pHc-sensitive cells, cells might accommodate their behaviors to variable systemic pH changes, and abnormality of this environmental pH sensing and responding might be related to disease onset and progression of metabolic diseases (46, 59, 60). Indeed, in our recent study, glucose challenge-induced pHc alkalization in pancreatic β cells is blunted in HFD-induced T2DM, and the abnormality of this high glucose-triggered pHc increase is a key mechanism for insulin processing defects during the development of T2DM (33). Type 2 alveolar cells are important for lung development and regeneration, and pigment epithelial cells are essential for normal homeostasis in the retina, while the functions of both cell types are severely affected in HFD overnutrition and T2DM (61, 62). In the future, it will be interesting to study whether pHc abnormalities in these cell types also play a role in the pathogenesis of these cell types in metabolic disorders. We also observed divergent pHc dynamics in tumor-infiltrating macrophages *vs.* T cells. Future studies should examine whether macrophage acidification and T cell alkalization contribute to immune activation within the TME and whether targeting pHc in these immune cells could serve as a novel and efficient way for anti-tumor immunotherapy.

Oral sodium bicarbonate or intravenous drip has been used in the treatment of a wide spectrum of diseases (33, 63, 64). Alkalization therapy plus chemotherapy can significantly improve the median survival of patients. Our previous research has also shown that alkaline water supply ameliorates pHc acidification in pancreatic β cells and insulin processing defects in HFD-treated mice (33). Detecting pHc in cells under physiological or pathophysiological conditions and understanding the pHc biology may provide a theoretical basis for the diagnosis of acid-base disturbances as well as clinical interventions.

Our study encompasses some limitations, which may serve as directions for future investigation. We have mapped pHc of major cell types in mice under healthy and diseased conditions. Apparently, there is a large amount of work remaining in order to dissect out the underlying mechanisms governing pHc maintenance and sensing, downstream cellular adaptations upon pHc changes, and pathophysiological implications under the circumstance of pHc misregulation in disease states.

## Experimental procedures

### Animal studies

All animal experiments were approved by the Laboratory Animal Research Center, Tongji University. All procedures involving animals were carried out in compliance with the Guide for the Care and Use of Laboratory Animals, and ethical approval was granted by the Ethics Committee, Tongji University (approval number: 2020YANYUSHEN093). Mice were maintained on a C57BL/6J background and housed on a 12 h light/dark cycle.

### Cell culture and generation of stable cell lines

HEK293FT and HeLa cells were regularly cultured in Dulbecco's Modified Eagle's Medium (Corning) supplemented with 10% fetal bovine serum (ExCell Bio) in 5% CO_2_ at 37 °C. EGFP-Smad5 DNA sequence was constructed into the pLenti vector for constitutive expression, and lentiviruses were packaged in HEK293FT cells as we previously described (65). Viruses were then concentrated by ultracentrifugation and used for making stable cell lines in HEK293FT cells and HeLa cells. The *Mycoplasma* test was regularly performed twice per week.

### Measurement of pHc in cultured cells

pHc in cultured cells was measured with the 2′,7′-bis-(2-carboxyethyl)-5-carboxyfluorescein acetoxymethyl ester (BCECF-AM) probe as previously described (24, 66). Wild type HEK293FT cells and HeLa cells were plated on laminin-coated 96-well plates at a density of 5000 cells per well. 36 h after seeding, cells were washed twice with Hank's balanced salt solution and then incubated with 1 μM BCECF-AM in the same buffer for 20 min. In one group, cells were washed twice with buffer A (145 mM KCl, 1 mM MgCl_2_, 1 mM CaCl_2_, 10 mM glucose and 10 mM HEPES) with a pH of 6.0, 6.4, 6.8, 7.2, 7.6 or 8.0 followed by 5 μM Nigericin (Selleck) treatment in buffer A with same pH. In another group, cells were washed twice with buffer B (140 mM NaCl, 5 mM KCl, 1 mM MgCl_2_, 1 mM CaCl_2_, 10 mM glucose and 10 mM HEPES) with a pH of 6.0, 6.4, 6.8, 7.2, 7.6 or 8.0 without Nigericin treatment. Both groups were incubated at 37 °C for 40 min in the absence of CO_2_ supply in order to maintain a stable preset pH in the incubation buffer. BCECF fluorescence of each well was subsequently measured with a microplate reader (Tecan Spark) at two excitation wavelengths (440/20 nm and 490/20 nm) both collected at a collection window of 530/20 nm. In the Nigericin treatment group, pHc was equilibrated with pHe designed and a standard curve between pHc and BCECF fluorescence was therefore established. Under the same buffering system and designed pHe, HEK293FT cells and HeLa cells with stable expression of eGFP-Smad5 were subjected to measurement of eGFP-Smad5 nucleocytoplasmic ratio without Nigericin treatment and the functions between the nucleocytoplasmic ratio of eGFP-Smad5 and pHc were constructed.

### Generation of pHc reporter transgenic mice

The CAG promoter-eGFP-Smad5 expression cassette flanked by 5′ and 3′ Rosa26 locus homologous arms was constructed in the pTight vector (27) and microinjected into zygotes with a C57BL/6 background together with Rosa 26 targeting sgRNA and Cas9 expression vectors. After microinjection, zygotes were transferred to pseudopregnant C57BL/6 mice. The primers used for genotyping are listed in Table S1.

### Western blotting analysis

Western blot analysis of protein expression was carried out as previously described (27). Total proteins were isolated from tissues by homogenization in lysis buffer (50 mM Tris-HCl, pH 8.0, 150 mM NaCl, 1% NP-40, 0.5% sodium deoxycholate, 0.1% SDS, and complete protease inhibitor cocktail (Roche)). Protein concentration was detected by Pierce BCA Protein Assay Kit. Primary antibodies used were β-Actin (1:2000, mouse IgG, Sigma, A5316), Smad5 (1:2000, rabbit IgG, Cell signaling, 9517), P-Smad1/5/8 (1:1000, rabbit IgG, Cell signaling, 9511), β-Tubulin (1:1000, mouse IgG, Sigma, T5201), Lamin A/C (1:1000, mouse IgG, Santa Cruz), GFP (1:2000, rabbit IgG, Invitrogen, A6455), and GAPDH (1:4000, rabbit IgG, Bioworld, AP0063).

### Nuclear and cytoplasmic fractionation

HEK293FT cells and HeLa cells with stable expression of eGFP-Smad5 were cultured in 10 cm dish for 48 h. Cells were washed twice with buffer B with a pH of 6.0, 6.4, 6.8, 7.2, 7.6, or 8.0, and then incubated at 37 °C for 40 min in the absence of a CO_2_ supply in order to maintain a stable preset pH in the incubation buffer. Cells were then permeabilized using 3 ml of buffer B with a pH of 6.0, 6.4, 6.8, 7.2, 7.6, or 8.0 containing 160 μg/ml digitonin for 3 min. Nuclei were pelleted by centrifugation at 300*g* for 5 min, and supernatants containing cytoplasmic proteins were collected.

### Immunohistochemistry

pHc reporter mice were quickly anesthetized with avertin (600 mg/kg) and immediately perfused intracardially with 4% paraformaldehyde (PFA) prewarmed at 37 °C. Tissues were removed and postfixed in the same fixative for 2 to 4 h at room temperature, which were then replaced with 20% and 30% sucrose overnight. Tissues were fast-frozen in cryo-embedding compound and cut into 16-μm sections. Sections were subjected to antigen retrieval followed by blocking (0.4% Triton X-100, 10% donkey serum) for 1 h at room temperature. Primary antibodies were applied in staining buffer (0.2% Triton X-100, 5% donkey serum) with overnight incubation at 4  °C. The next day, sections were washed and incubated in secondary antibodies (1:1000, Jackson ImmunoResearch, West Grove, USA) for 1 h at room temperature. Sections were counterstained with DNA dye bisbenzimide (Hoechst 33,258) and mounted with Fluoromount-G (SouthernBiotech, Birmingham, USA). The antibodies used were GFP (1:500, chicken IgY, Aves, GFP-1020), β-catenin (1:300, rabbit IgG, Abcam, ab32572), β-catenin (1:300, mouse IgG, BD, 610,154), Rpe65 (1:300, rabbit IgG, Abcam, ab231782), Ager (1:300, rabbit IgG, Abcam, ab216329), Rbpms (1:100, rabbit IgG, Proteintech, 10073-1-AP), Sftpc (1:500, rabbit IgG, Proteintech, 10074-1-AP), Hopx (1:200, mouse IgG, Santa Cruz, sc-398703), Alb (1:300, rabbit IgG, GeneTex, GTX102419), F4/80 (1:500, rabbit IgG, Abclonal, A23788), H^+^/K^+^ ATPase β (1:200, mouse IgG, Santa Cruz, sc-374094), Tff2 (1:500, rabbit IgG, Proteintech, 13681-1-AP), Na^+^/K^+^ ATPase (1:200, rabbit IgG, Abclonal, A11683), Mist1 (1:200, mouse IgG, Santa Cruz, sc-166181), Insulin (1:2000, rabbit IgG, CST, 3014) and Glucagon (1:2000, rabbit IgG, Abcam, ab92517), CD3 (1:300, mouse IgG, Servicebio, GB12014), CD4 (1:300, rabbit IgG, Servicebio, GB15064), and CD8 (1:300, rabbit IgG, Servicebio, GB15068).

### Cell-line-derived xenograft model

2.5 × 10^5^ B16 melanoma cells were subcutaneously injected into the pHc reporter mice at a volume of 100 μl. 21 days after tumor inoculation, the pHc reporter mice were quickly anesthetized with avertin and perfused intracardially with 4% PFA prewarmed at 37 °C. Tumors were removed and postfixed in the same fixative for 30 min at 37 °C and an additional 6 to 8 h at room temperature for sectioning and immunohistology analyses.

### Measurement of pHc *in vivo*

After immunofluorescence staining, images were captured using a Leica TCS SP8 (Leica Microsystems) confocal laser-scanning microscope. Confocal planes were scanned every 0.8 μm with 4 to 6 scans. Cell boundary was characterized by staining of plasma membrane conjugated β-catenin or cytoplasm, predominantly distributed cell type-specific proteins, and the nucleus was counterstained with Hoechst 33,258. The fluorescence intensity of eGFP-Smad5 in the nucleus and cytoplasm was calculated using ImageJ software, and the nucleocytoplasmic ratio of eGFP-Smad5 was calculated. pHc of each individual cell was calculated based on the following mathematical model: y = 8.20631 − 1.28918x + 0.35457x^2^ − 0.03241x^3^, where y represents pHc and x represents nucleocytoplasmic ratio of eGFP-Smad5. For systemic acidification and alkalization studies, male pHc reporter mice aged at 6 to 8 weeks were treated with an acute intraperitoneal (i.p.) injection of 15% lactic acid or 7% sodium bicarbonate at pH 9.5 (10 μl per g body weight).

### Magnetic resonance imaging

Magnetic resonance imaging (MRI) experiment was carried out as previously described (22, 34). Animal MRI studies were performed in Bruker 9.4-T small animal MRI scanner (Bruker Biospec 94/20 USR; Bruker Biospin, Germany). The bore of the magnet is 16 cm. Before chemical exchange saturation transfer (CEST) experiments, applied magnetic field (B1) and main magnetic field (B0) field maps for the locations of interest were generated. Water saturation shift referencing (WASSR) sequence was used to perform B0 offset correction on CEST data. WASSR sequence: echo time (TE) = 32.64 ms, repetition time (TR) = 1000 ms, average = 3, rare factor (RF) = 16, slices thickness = 1 mm, image size = 90 × 90 mm^2^, field of view (FOV) = 30 × 30 mm^2^, resolution = 0.33 × 0.33, B1 amplitude = 0.4 μT, saturation frequency = −2.5 to 2.5 ppm (51 steps), total 4 min and 15 s. CEST images were acquired using a rapid acquisition with relaxation enhancement (RARE) sequence (slice thickness = 2 mm, TR = 40 ms, TE = 0.316 ms, average = 1, image size = 128 × 128 mm^2^, FOV = 20 × 20 mm^2^, resolution = 0.15 × 0.15, B1 amplitude = 10 μT. The saturation frequency was expressed in ppm relative to water and included the following ranges: −10 ppm, −0.4–0.4 ppm (0.1 ppm step), 1.6 to 4.2 ppm (0.1 ppm step), 5.6 to 6.4 ppm (0.1 ppm step), and 10 ppm. The remaining steps were spaced at 0.2 ppm intervals, for a total of 114 steps). The total scan duration was 30 min and 33 s.

$$AACID=\frac{M_{Z}\left(3.50p.p.m.\right)\times \left(M_{Z}\left(6.0p.p.m.\right)-M_{Z}\left(2.75p.p.m.\right)\right)}{M_{Z}\left(2.75p.p.m.\right)\times \left(M_{Z}\left(6.0p.p.m.\right)-M_{Z}\left(3.50p.p.m.\right)\right)}$$

The resulting relation obtained by liver tissue homogenate pH buffer calibration described in equation (pH = 57.843 × AACID^3^ − 235.24 × AACID^2^ + 300.9 × AACID − 109.99) was used to produce quantitative pH maps *in vivo*.

### Liver pHc measurement with BCECF probe

After tail vein injection of 200 μl BCECF-AM (25 μM) for 40 min, mice were quickly anesthetized with avertin, and a drip of 50 μl of BCECF-AM (50 μM) was applied on the surface of the liver. After 10 min of incubation, the liver was rinsed three times with prewarmed physiological saline, and BCECF fluorescence was measured with a confocal microscope (Leica TCS SP8) at two excitation wavelengths (405 nm and 488 nm), both collected at a collection window of 520 to 540 nm. For systemic acidification, after tail vein injection of BCECF-AM, mice were i.p. injected with 15% lactic acid (10 μl per g body weight). The ratio of 488 nm/405 nm was calculated using ImageJ software.

### Rotarod test

Mice were trained for three sequential days on the rotarod. Each daily practice session consisted of placing the subject on the rotarod at a slow rotational speed (5 rpm) for 10 min. During testing on day 4, mice were placed on an accelerating rotarod from 5.0 to 40.0 rpm for 40 s. The latency to fall was measured.

### Measurement of homogenate pH

Measurement of homogenate pH was carried out as previously described (67). Fresh tissues were homogenized using a tissue homogenizer equipped with a conical pestle in ice-cold distilled H_2_O (1 ml per 100 mg tissue). Homogenate pH was measured using a pH meter (InLab Micro, Mettler Toledo) after a three-point calibration at pH 4.01, pH 7.00, and pH 9.21.

### GTT and ITT assays

For the glucose tolerance test (GTT), mice fasted for 16 h were i.p. injected with 1 g glucose per kg body weight. Blood glucose was measured at 0, 15, 30, 60, 90, and 120 min after glucose challenge with a Roche glucometer. An insulin tolerance test (ITT) was performed following a 6-hour fast. Mice were i.p. injected with 0.75 U per kg body weight of recombinant human insulin prepared in sterile PBS. Tail tip blood was then collected at 0, 15, 30, 60, 90, and 120 min to measure glucose levels.

### Statistical analysis

Data are presented as mean ± SD. An unpaired two-tailed Student's *t* test was used for statistical analysis for two groups. One-way ANOVA was used for multiple comparisons. Statistical significance is considered at *p* values below 0.05. ∗*p* < 0.05; ∗∗*p* < 0.01, ∗∗∗*p* < 0.001, and ∗∗∗∗*p* < 0.0001.

## Data availability

The authors declare that the data underlying the findings of this study are available within the article and its Supporting Information and are available upon request.

## Supporting information

This article contains supporting information.

## Conflict of interest

The authors declare that they have no conflicts of interest with the contents of this article.
