# Supplementary material for: Mapping cellular cytosolic pH in vivo under physiological and pathophysiological conditions
Source: J Biol Chem. 2025 Aug 28;301(10):110637. doi: 10.1016/j.jbc.2025.110637 (PMC12494554; doi:10.1016/j.jbc.2025.110637)
Supplement: Supporting Information [file mmc1.pdf]

Mapping cellular cytosolic pH *in vivo* under physiological and pathophysiological conditions

Shuwei Zhang[1,2,4,5,6,‡], Xiaoxin Hu[1,2,4,5,6,‡], Bowen Zhang[1,2,4,5,6], Jingxuan Liu[1,2,4,5,6], Hexi Feng[1,2,4,5,6], Caiying Liu[1,2,4,5,6], Yi Hui[1,2,4,5,6], Yujiang Fang[1,2,4,5,6*], Ling Liu[1,2,4,5,6*], Xiaoqing Zhang[1,2,3,4,5,6,7*]

[1]Translational Research Institute of Brain and Brain-Like Intelligence, Shanghai Fourth People's Hospital, School of Medicine, Tongji University, Shanghai, China

[2]Translational Medical Center for Stem Cell Therapy, Shanghai East Hospital, School of Medicine, Tongji University, Shanghai, China

[3]Key Laboratory of Spine and Spinal Cord Injury Repair and Regeneration of Ministry of Education, School of Medicine, Tongji University, Shanghai, China

[4]Shanghai Institute of Stem Cell Research and Clinical Translation, Shanghai, China

[5]Key Laboratory of Neuroregeneration of Shanghai Universities, School of Medicine, Tongji University, Shanghai, China

[6]Stem Cell Research Center, School of Medicine, Tongji University, Shanghai, China

[7]Clinical Center for Brain and Spinal Cord Research, Tongji University, Shanghai, China

[‡] These authors contributed equally to this work.

*To whom correspondence should be addressed.

Contents

Figure SF1-SF21
Table S1.

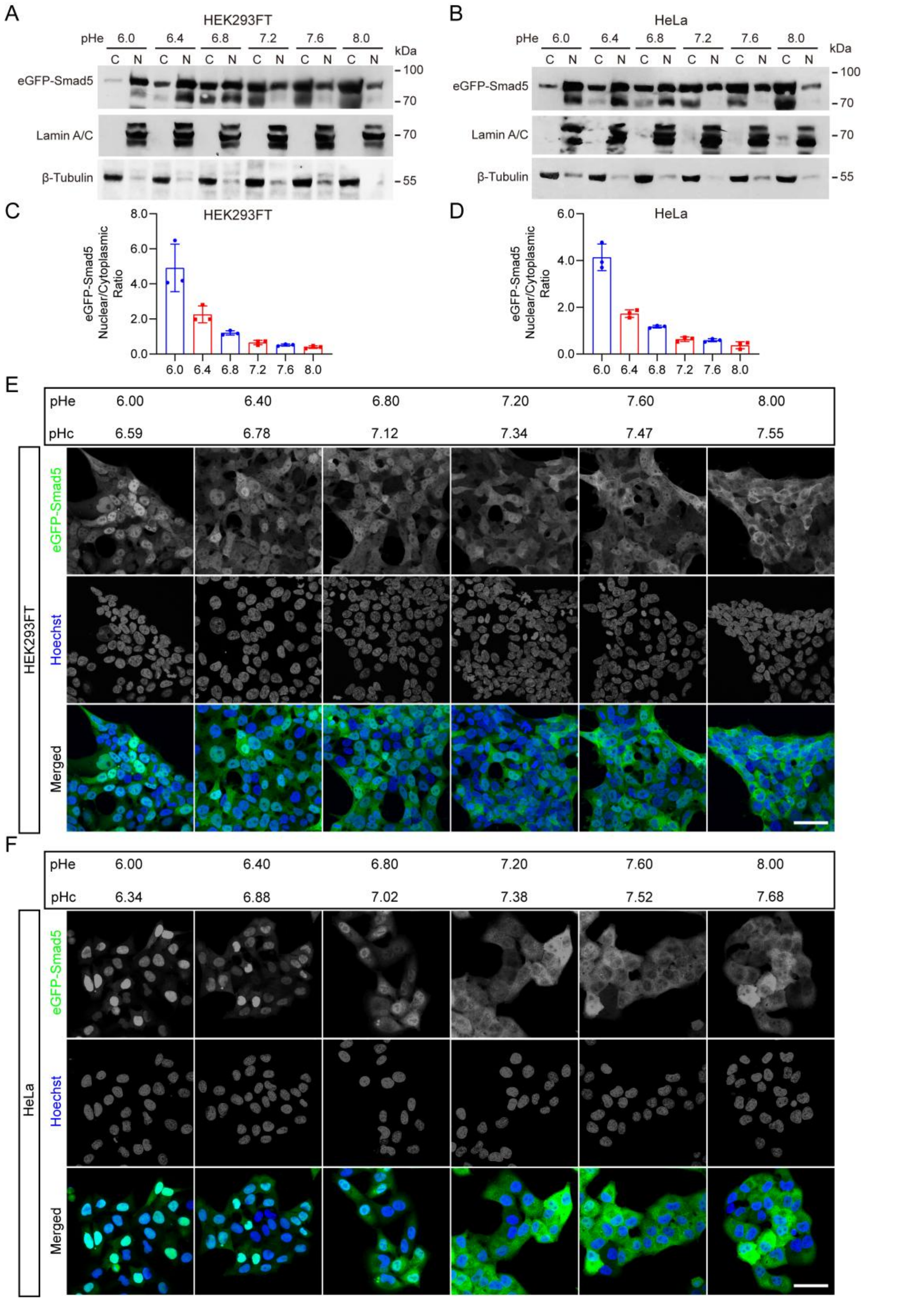

Figure SF1. The correlation of nucleocytoplasmic distribution of eGFP-Smad5, pHe and calculated pHc. Related to **Figure 1**.

A and B, western blot studies show eGFP-Smad5 nucleocytoplasmic shuttling upon

pHe alkalization in HEK293FT cells (A) and HeLa cells (B).

C and D, quantification of eGFP-Smad5 nucleocytoplasmic ratio by densitometry in (A) and (B). Data are presented as mean ± SD; n = 3 for all groups.

E and F, representative immunofluorescence staining of eGFP-Smad5 (green) and Hoechst 33258 (blue) in HEK293FT cells (E) and HeLa cells (F) under variable pHe and pHc without Nigericin treatment. Scale bars, 50 μm.

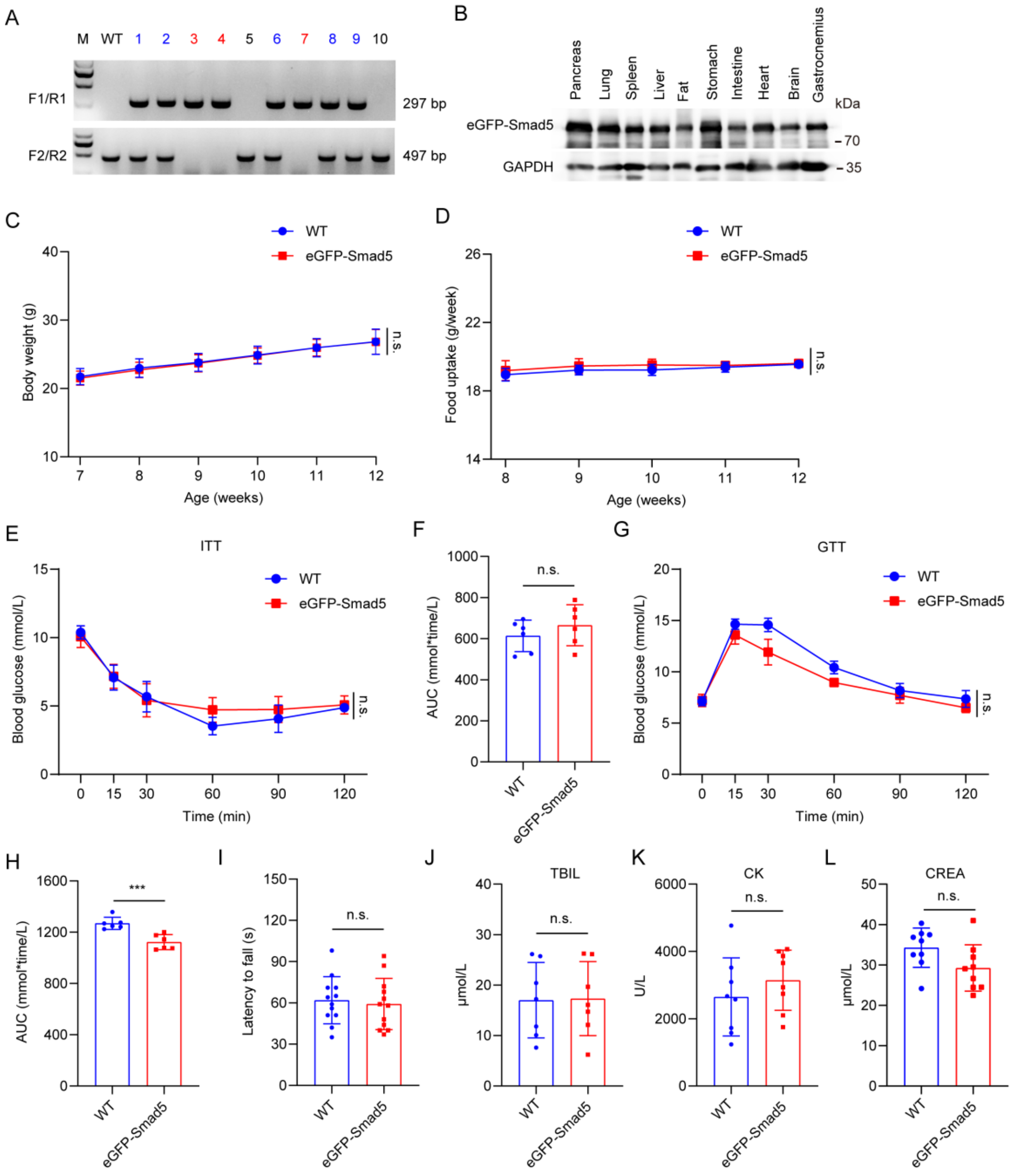

Figure SF2. Transgenic pHc reporter mice are phenotypically normal. Related to

Figure 2.

A, genomic DNA PCR validation of pHc reporter mice. Primer set F1/R1 amplicons represent the eGFP-Smad5 and primer set F2/R2 amplicons represent the wild type allele. Red numbers indicate eGFP-Smad5 homozygous mice, blue numbers indicate eGFP-Smad5 heterozygous mice.

B, western blot analysis reveals that eGFP-Smad5 is expressed in all tissues examined.

C, body weight changes in WT mice and eGFP-Smad5 mice. Data are presented as mean ± SD; statistical analysis was performed using one-way ANOVA and no significant difference was observed; n = 6 for both groups.

D, the average weekly food uptake per WT and eGFP-Smad5 mice. Data are presented as mean ± SD; statistical analysis was performed using one-way ANOVA and no significant difference was observed; n = 6 for both groups.

E, insulin tolerance test in WT mice and eGFP-Smad5 mice. Data are presented as mean ± SD; statistical analysis was performed using one-way ANOVA and no significant difference was observed; n = 6 for both groups.

F, AUC in (E). Data are presented as mean ± SD; n.s., no significant difference, unpaired two-tailed *t* test; n = 6 for both groups.

G, glucose tolerance test in WT mice and eGFP-Smad5 mice. Data are presented as mean ± SD; statistical analysis was performed using one-way ANOVA and no significant difference was observed; n = 6 for both groups.

H, AUC in (G). Data are presented as mean ± SD; *** $p < 0.001$, unpaired two-tailed *t* test; n = 6 for both groups.

I, the latency to fall from an accelerating rotarod test was similar in WT mice and eGFP-Smad5 mice. Data are presented as mean ± SD; n.s., no significant difference, unpaired two-tailed *t* test; n = 12 for both groups.

J, serum total bilirubin (TBIL, indicator for liver function) levels in WT mice and eGFP-Smad5 mice. Data are presented as mean ± SD; n.s., no significant difference, unpaired two-tailed *t* test; n = 7 for both groups.

K, serum creatine kinase (CK, indicator for heart function) levels in WT mice and eGFP-Smad5 mice. Data are presented as mean ± SD; n.s., no significant difference, unpaired two-tailed *t* test; n = 8 for all groups.

L, serum creatinine (CREA, indicator for kidney function) levels in WT mice and eGFP-Smad5 mice. Data are presented as mean ± SD; n.s., no significant difference, unpaired two-tailed *t* test; n = 9 for both groups.

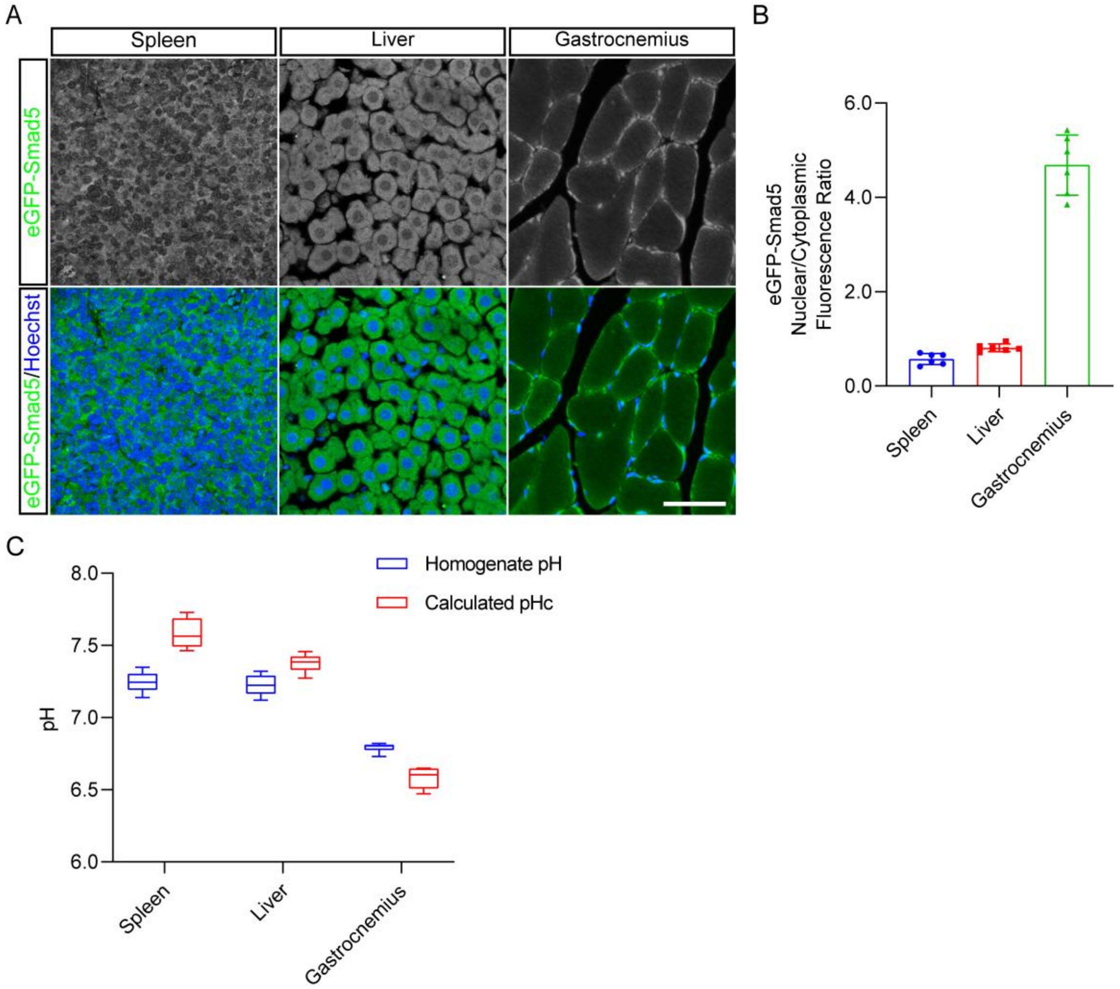

Figure SF3. eGFP-Smad5 nucleocytoplasmic ratio calculated pHc coincides with homogenate pH. Related to **Figure 2**.

A, representative immunofluorescence staining of eGFP-Smad5 (green) and Hoechst 33258 (blue) in spleen, liver and gastrocnemius of pHc reporter mice. Scale bar, 50 µm.

B, eGFP-Smad5 nucleocytoplasmic fluorescence ratio quantification in (A). Data are presented as mean ± SD; n = 6 mice.

C, the calculated pHc in cells of spleen, liver and gastrocnemius highly coincides with their homogenate pH. Data are presented as mean ± SD; n = 6 mice.

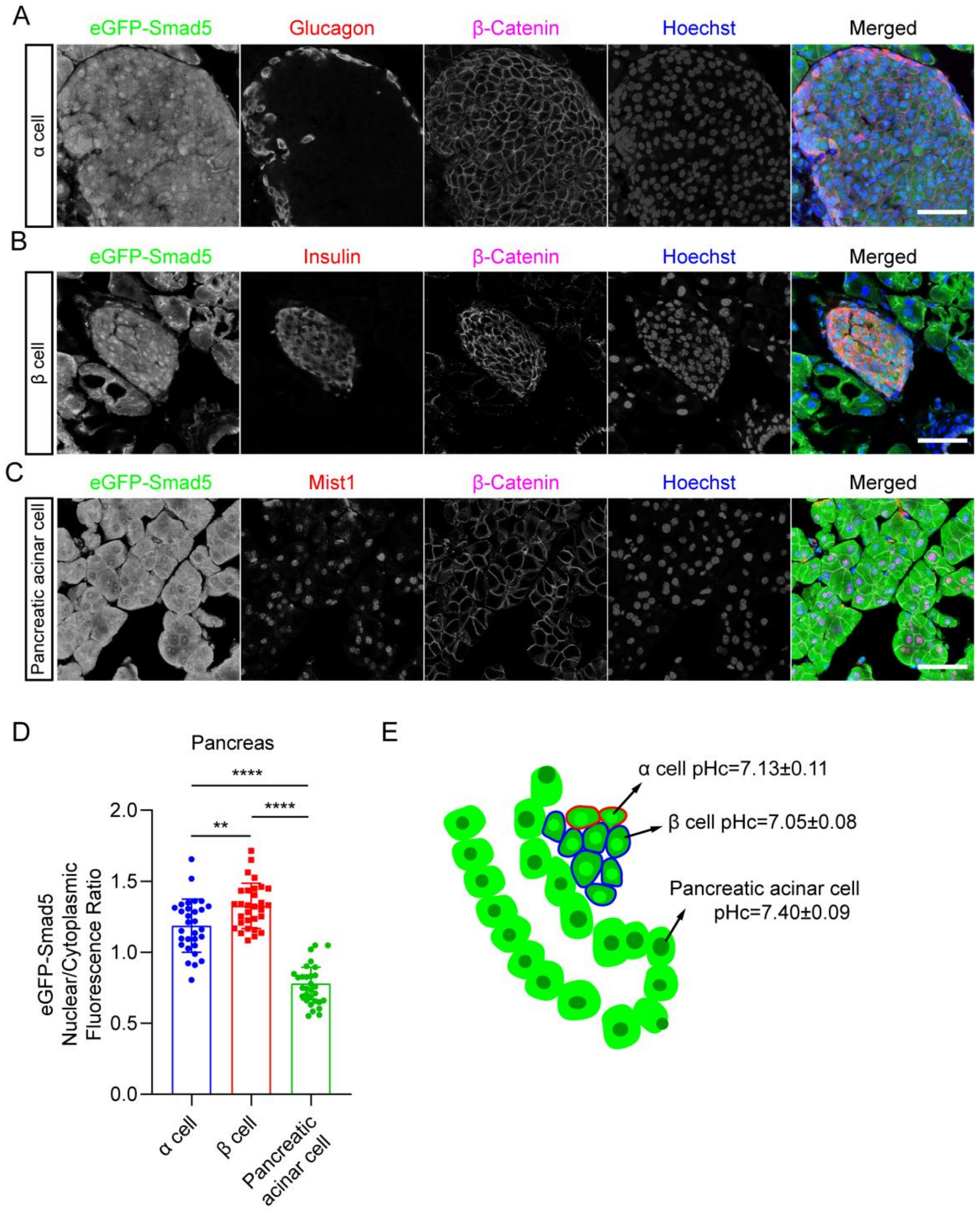

Figure SF4. pHc of major cell types in mice pancreas under baseline conditions.

Related to **Figure 3**.

A, B, and C, representative immunofluorescence staining of eGFP-Smad5 (green), cell type specific markers (Glucagon for α cells (A); Insulin for β cells (B); Mist1 for pancreatic acinar cells (C), red), β-catenin (labeling plasma membrane boundary,

magenta), and Hoechst 33258 (blue) in the pancreas of pHc reporter mice. Scale bars, 50 μm.

D, eGFP-Smad5 nucleocytoplasmic ratio quantification of α cells, β cells, and pancreatic acinar cells in (A, B, and C). Data are presented as mean ± SD; $^{**}p < 0.01$, **** $p < 0.0001$, unpaired two-tailed *t* test; n = 30 cells for all groups.

E, the schematic diagram of eGFP-Smad5 nucleocytoplasmic distribution and pHc in α cells, β cells and pancreatic acinar cells in pancreas.

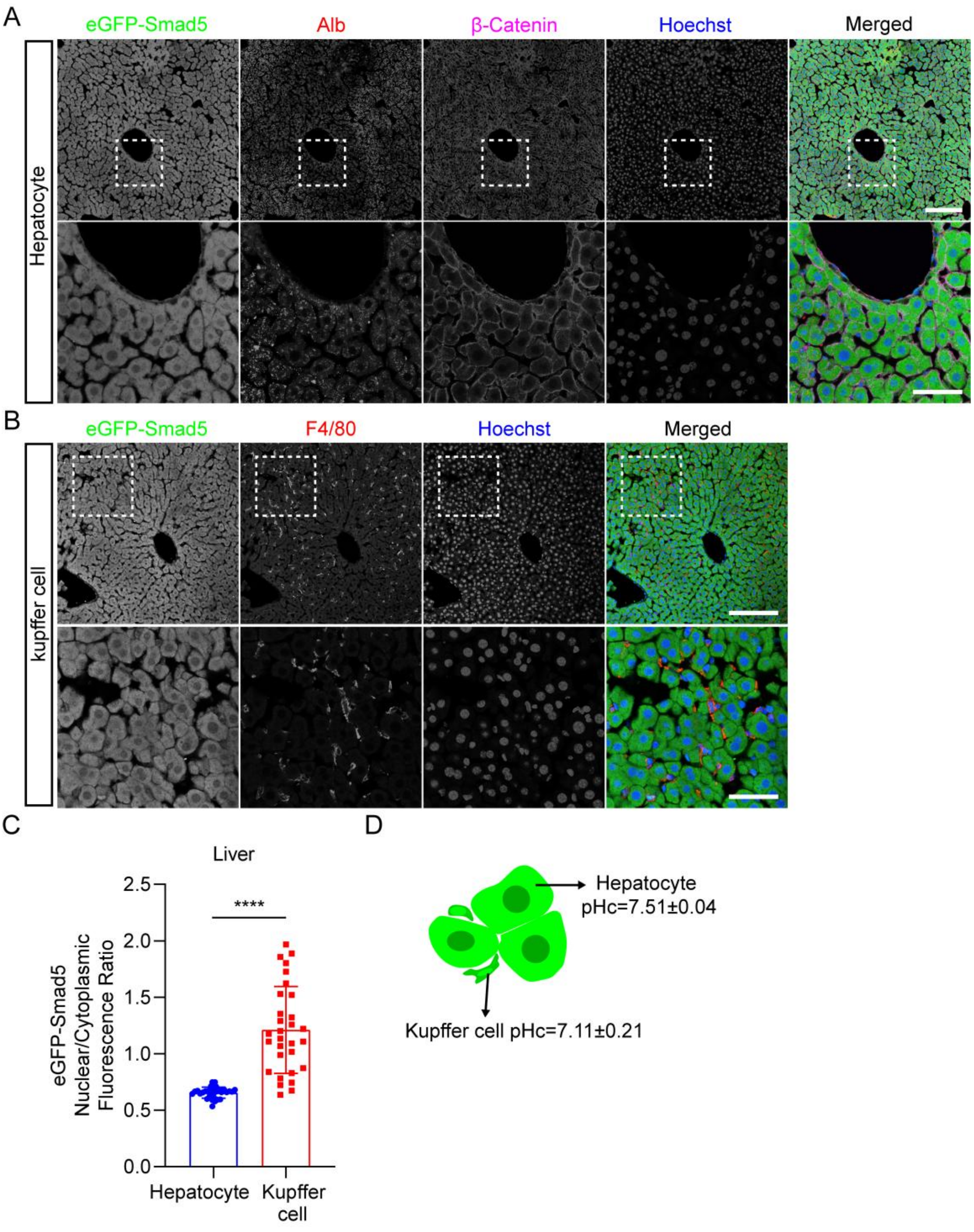

Figure SF5. pHc of major cell types in mice liver under baseline conditions. Related to **Figure 3**.

A and B, representative immunofluorescence staining of eGFP-Smad5 (green), cell type specific markers (Alb for hepatocytes (A); F4/80 for Kupffer cells (B), red), β-catenin (magenta), and Hoechst 33258 (blue) in the liver of pHc reporter mice. White

dotted line outlines the regions at higher magnification at the lower panel. Scale bars, 150 µm for low magnification; 50 µm for high magnification.

C, eGFP-Smad5 nucleocytoplasmic ratio quantification of hepatocytes and Kupffer cells in (A and B), respectively. Data are presented as mean ± SD; **** $p < 0.0001$, unpaired two-tailed t test; n = 30 cells for both groups.

D, the schematic diagram of eGFP-Smad5 nucleocytoplasmic distribution and pHc in hepatocytes and Kupffer cells in liver.

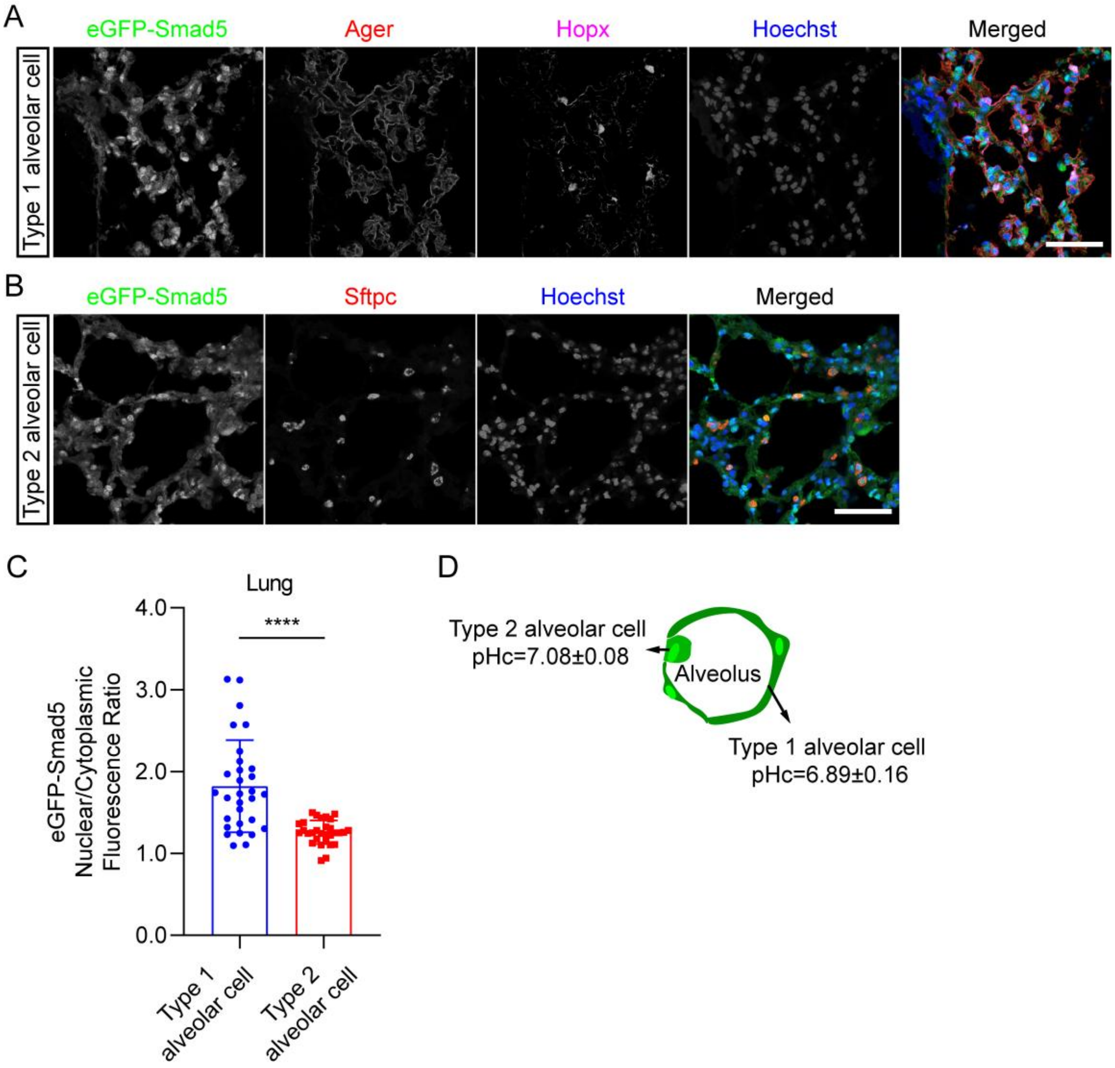

Figure SF6. pHc of major cell types in mice lung under baseline conditions. Related to **Figure 3**.

A and B, representative immunofluorescence staining of eGFP-Smad5 (green), cell type specific markers (Ager and Hopx for type 1 alveolar cells, red and magenta, respectively (A); Sftpc for type 2 alveolar cells, red (B)) and Hoechst 33258 (blue) in the lung of pHc reporter mice. Scale bars, 50 μm.

C, eGFP-Smad5 nucleocytoplasmic ratio quantification of type 1 alveolar cells and type 2 alveolar cells in (A and B), respectively. Data are presented as mean ± SD; **** $p < 0.0001$, unpaired two-tailed *t* test; n = 30 cells for both groups.

D, the schematic diagram of eGFP-Smad5 nucleocytoplasmic distribution and pHc in type 1 alveolar cells and type 2 alveolar cells in lung.

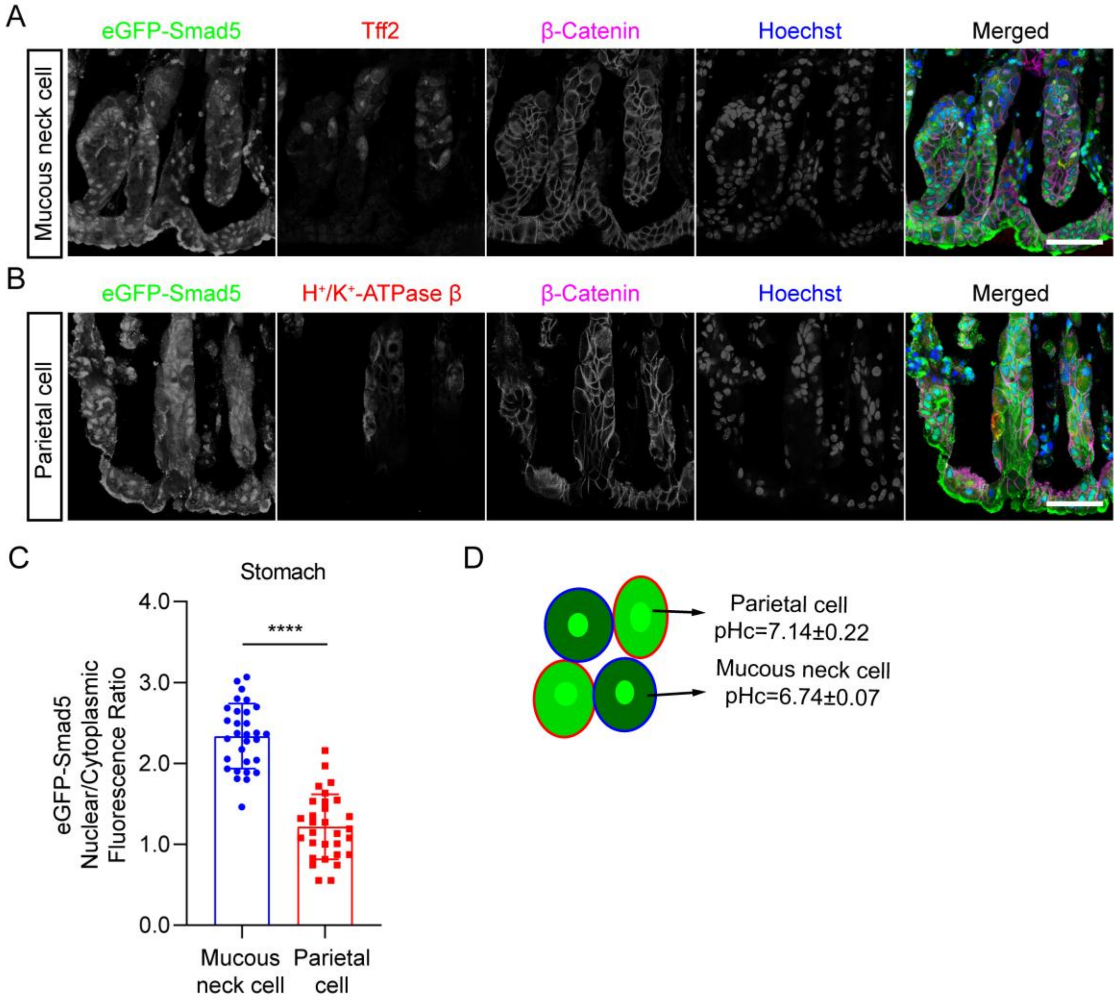

Figure SF7. pHc of major cell types in mice stomach under baseline conditions.

Related to **Figure 3**.

A and B, representative immunofluorescence staining of eGFP-Smad5 (green), cell type specific markers (Tff2 for mucous neck cell (A); H^{+}/K^{+}-ATPase β for parietal cell (B), red), β-catenin (magenta), and Hoechst 33258 (blue) in the stomach of pHc reporter mice. Scale bars, 50 μm.

C, eGFP-Smad5 nucleocytoplasmic ratio quantification of mucous neck cells and parietal cells in (A and B), respectively. Data are presented as mean ± SD; **** $p < 0.0001$, unpaired two-tailed *t* test; n = 30 cells for both groups.

D, the schematic diagram of eGFP-Smad5 nucleocytoplasmic distribution and pHc in mucous neck cells and parietal cells in stomach.

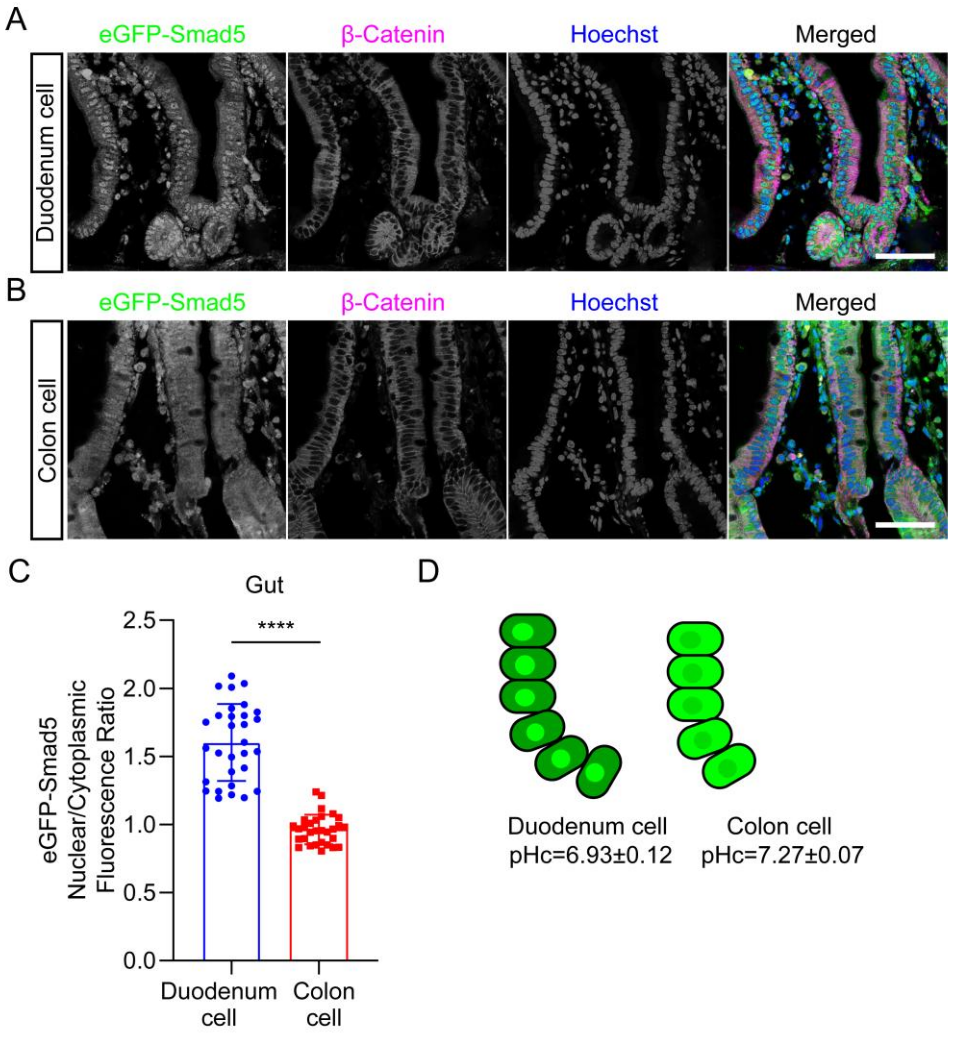

Figure SF8. pHc of mice gut cells under baseline conditions. Related to **Figure 3**.

A and B, representative immunofluorescence staining of eGFP-Smad5 (green), β-catenin (magenta), and Hoechst 33258 (blue) in duodenum cells (A) and colon cells (B) of pHc reporter mice. Scale bars, 50 μm.

C, eGFP-Smad5 nucleocytoplasmic ratio quantification of duodenum cells and colon cells in (A and B), respectively. Data are presented as mean ± SD; **** $p < 0.0001$, unpaired two-tailed *t* test; n = 30 cells for both groups.

D, the schematic diagram of eGFP-Smad5 nucleocytoplasmic distribution and pHc in duodenum cells and colon cells.

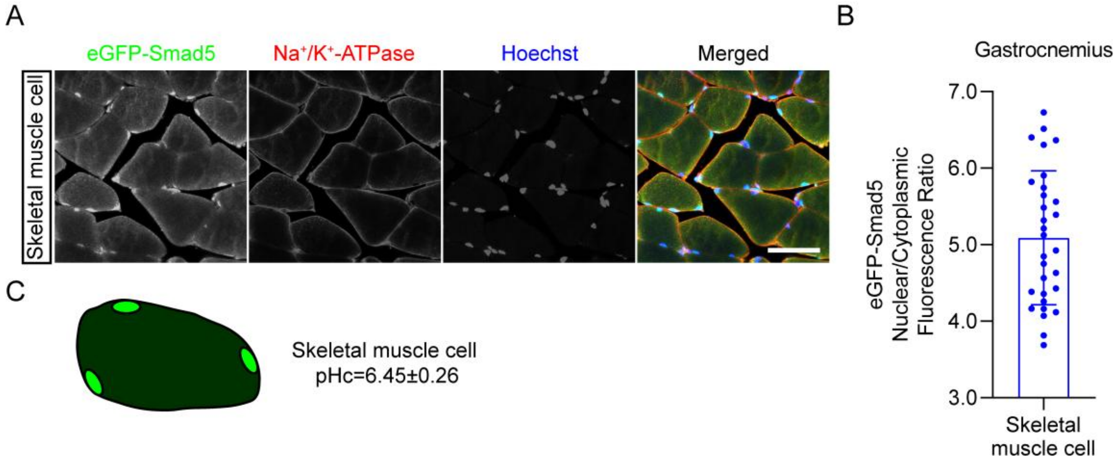

Figure SF9. pHc of mice skeletal muscle cells under baseline conditions. Related to **Figure 3**.

A, Representative immunofluorescence staining of eGFP-Smad5 (green), Na^+/K^+-ATPase (red), and Hoechst 33258 (blue) in skeletal muscle cells of pHc reporter mice. Scale bar, 50 μm.

B, eGFP-Smad5 nucleocytoplasmic ratio quantification of skeletal muscle cells in (A). Data are presented as mean ± SD; n = 30 cells.

C, The schematic diagram of eGFP-Smad5 nucleocytoplasmic distribution and pHc in skeletal muscle cells.

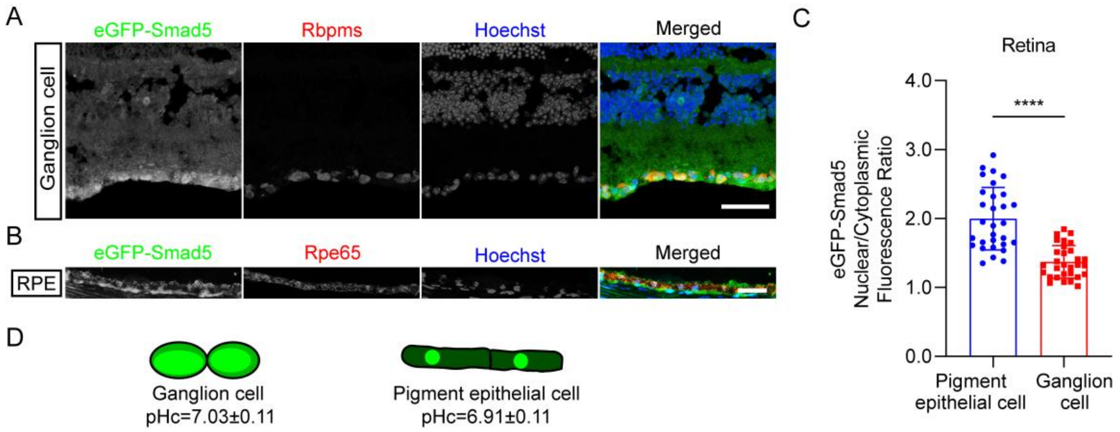

Figure SF10. pHc of major cell types in mice retina under baseline conditions. Related to **Figure 3**.

A and B, representative immunofluorescence staining of eGFP-Smad5 (green), cell type specific markers (Rbpms for ganglion cells (A); Rpe65 for retinal pigment epithelial cells (B), red), and Hoechst 33258 (blue) in the retina of pHc reporter mice. Scale bars in (A), 50 μm; in (B) 30 μm.

C, eGFP-Smad5 nucleocytoplasmic ratio quantification of ganglion cells and retinal pigment epithelial cells in (A and B), respectively. Data are presented as mean ± SD; **** $p < 0.0001$, unpaired two-tailed *t* test; n = 30 cells for both groups.

D, the schematic diagram of eGFP-Smad5 nucleocytoplasmic distribution and pHc in ganglion cells and retinal pigment epithelial cells in retina.

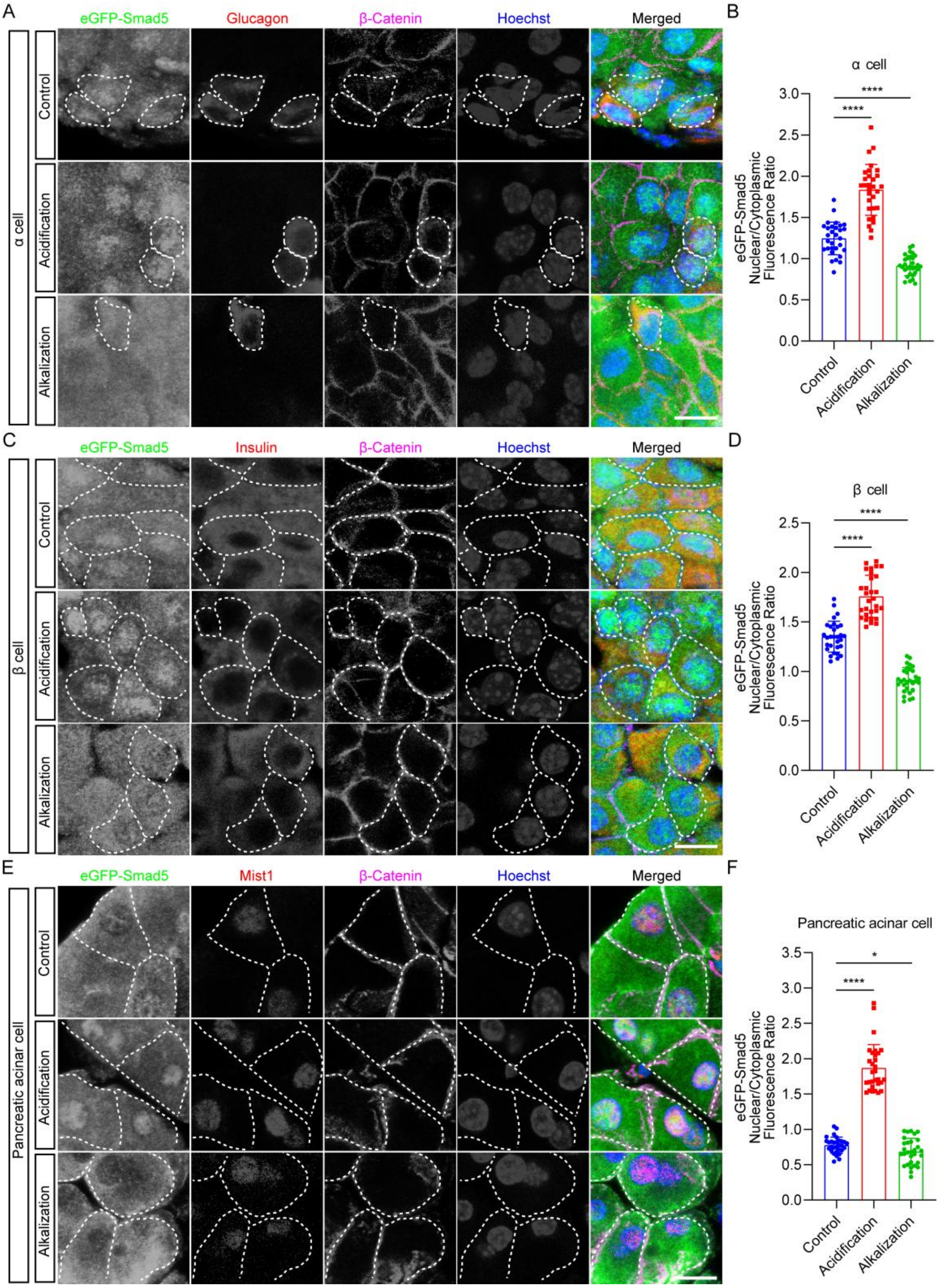

Figure SF11. pHc in pancreatic cells is sensitive to acute systemic acidification and alkalization challenges. Related to **Figure 4**.

A, representative immunofluorescence staining of eGFP-Smad5 (green), Glucagon (red), β-catenin (magenta), and Hoechst 33258 (blue) in α cells under systemic

acidification and alkalization conditions. Scale bar, 10 μm.

B, eGFP-Smad5 nucleocytoplasmic ratio quantification of α cells under systemic acidification and alkalization conditions in (A). Data are presented as mean ± SD; **** $p < 0.0001$, unpaired two-tailed *t* test; n = 30 cells for all groups.

C, representative immunofluorescence staining of eGFP-Smad5 (green), Insulin (red), β-catenin (magenta), and Hoechst 33258 (blue) in β cells under systemic acidification and alkalization conditions. Scale bar, 10 μm.

D, eGFP-Smad5 nucleocytoplasmic ratio quantification of β cells under systemic acidification and alkalization conditions in (C). Data are presented as mean ± SD; **** $p < 0.0001$, unpaired two-tailed *t* test; n = 30 cells for all groups.

E, representative immunofluorescence staining of eGFP-Smad5 (green), Mist1(red), β-catenin (magenta), and Hoechst 33258 (blue) in pancreatic acinar cells under systemic acidification and alkalization conditions. Scale bar, 10 μm.

F, eGFP-Smad5 nucleocytoplasmic ratio quantification of pancreatic acinar cells under systemic acidification and alkalization conditions in (E). Data are presented as mean ± SD; * $p < 0.05$, **** $p < 0.0001$, unpaired two-tailed *t* test; n = 30 cells for all groups.

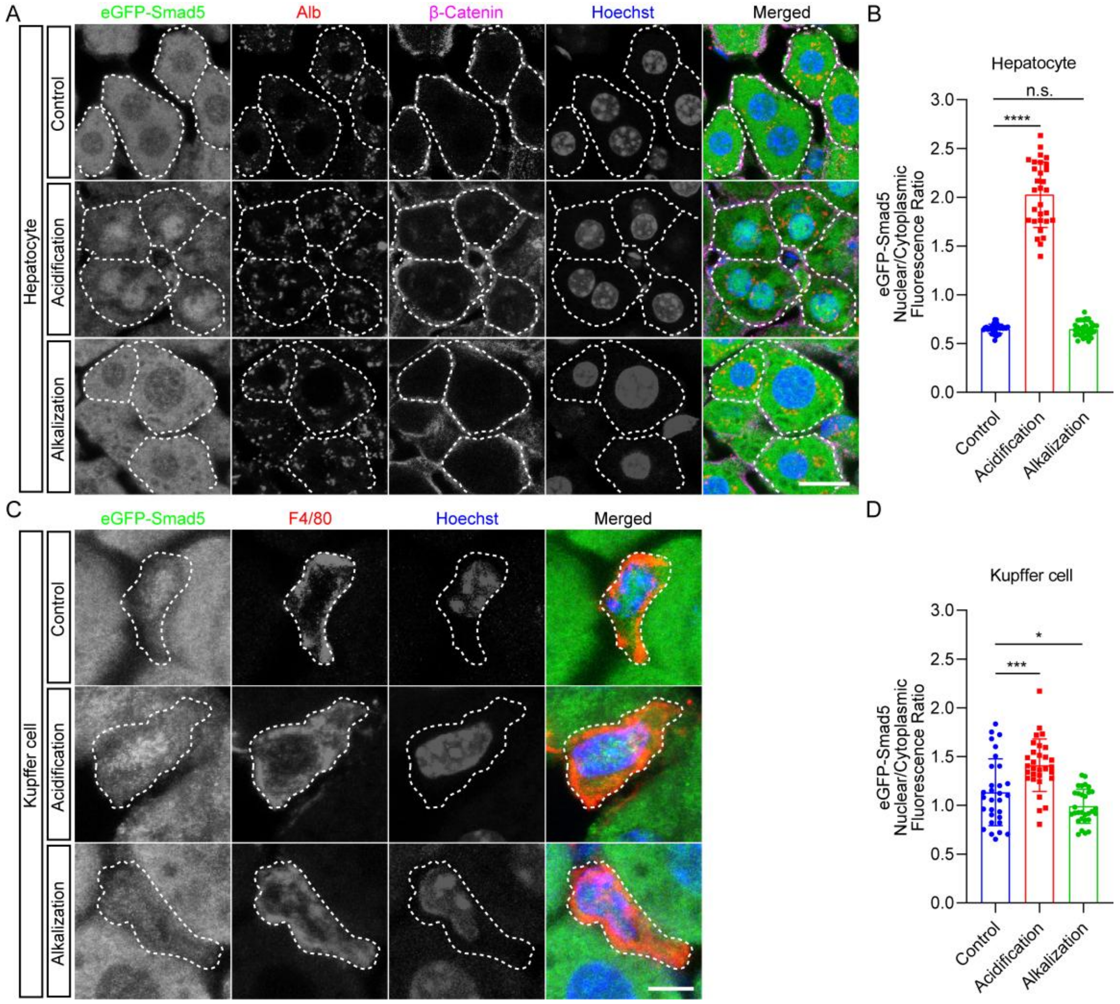

Figure SF12. pHc in liver cells is sensitive to acute systemic acidification and alkalization challenges. Related to **Figure 4**.

A, representative immunofluorescence staining of eGFP-Smad5 (green), Alb (red), β-catenin (magenta), and Hoechst 33258 (blue) in hepatocytes under systemic acidification and alkalization conditions. Scale bar, 12.5 μm.

B, eGFP-Smad5 nucleocytoplasmic ratio quantification of hepatocytes under systemic acidification and alkalization conditions in (A). Data are presented as mean ± SD; n.s., no significant difference, **** $p < 0.0001$, unpaired two-tailed *t* test; n = 30 cells for all groups.

C, representative immunofluorescence staining of eGFP-Smad5 (green), F4/80 (red),

and Hoechst 33258 (blue) in Kupffer cells under systemic acidification and alkalization conditions. Scale bar, 4 μm.

D, eGFP-Smad5 nucleocytoplasmic ratio quantification of Kupffer cells under systemic acidification and alkalization conditions in (C). Data are presented as mean ± SD; * $p < 0.05$, *** $p < 0.001$, unpaired two-tailed *t* test; n = 30 cells for all groups.

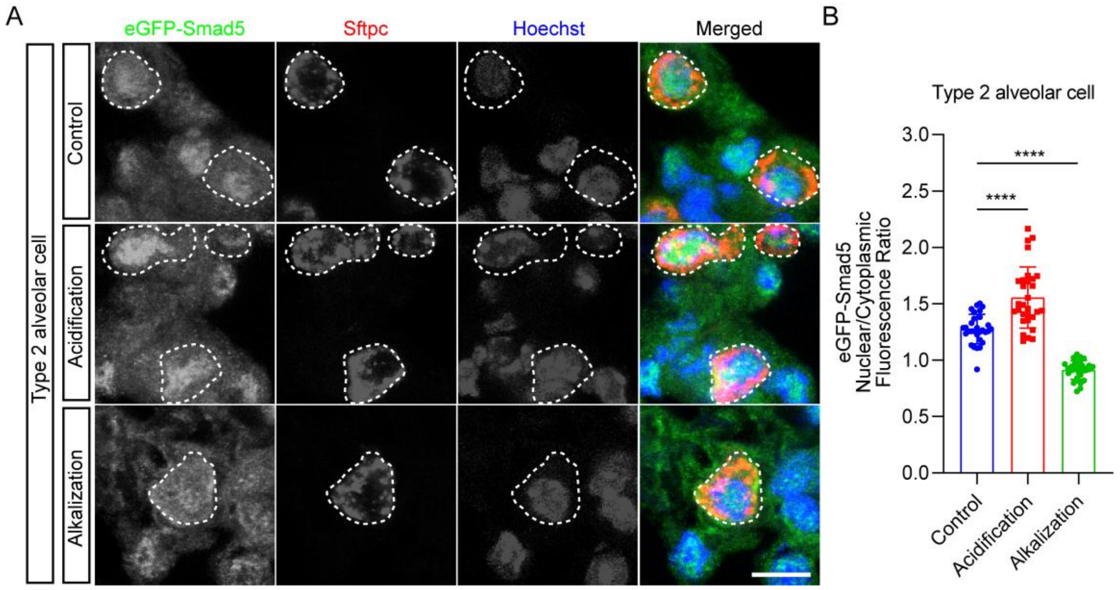

Figure SF13. pHc in type 2 alveolar cells is sensitive to acute systemic acidification and alkalization challenges. Related to **Figure 4**.

A, representative immunofluorescence staining of eGFP-Smad5 (green), Sftpc (red), and Hoechst 33258 (blue) in type 2 alveolar cells under systemic acidification and alkalization conditions. Scale bar, 6 μm.

B, eGFP-Smad5 nucleocytoplasmic ratio quantification of type 2 alveolar cells under systemic acidification and alkalization conditions in (A). Data are presented as mean ± SD; **** $p < 0.0001$, unpaired two-tailed *t* test; n = 30 cells for all groups.

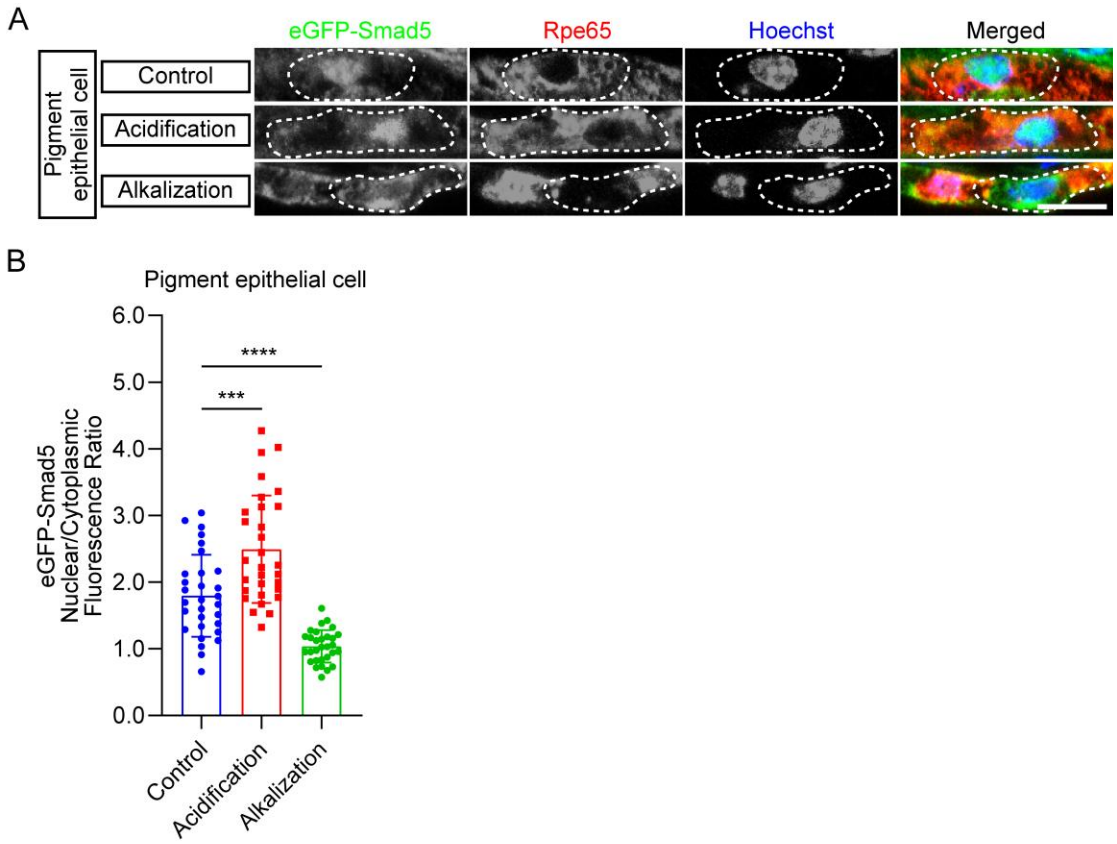

Figure SF14. pHc in retinal pigment epithelial cells is sensitive to acute systemic acidification and alkalization challenges. Related to **Figure 4.**

A, representative immunofluorescence staining of eGFP-Smad5 (green), Rpe65 (red), and Hoechst 33258 (blue) in retinal pigment epithelial cells under systemic acidification and alkalization conditions. Scale bar, 10 μm.

B, eGFP-Smad5 nucleocytoplasmic ratio quantification of retinal pigment epithelial cells under systemic acidification and alkalization conditions in (A). Data are presented as mean ± SD; *** $p < 0.001$, **** $p < 0.0001$, unpaired two-tailed *t* test; n = 30 cells for all groups.

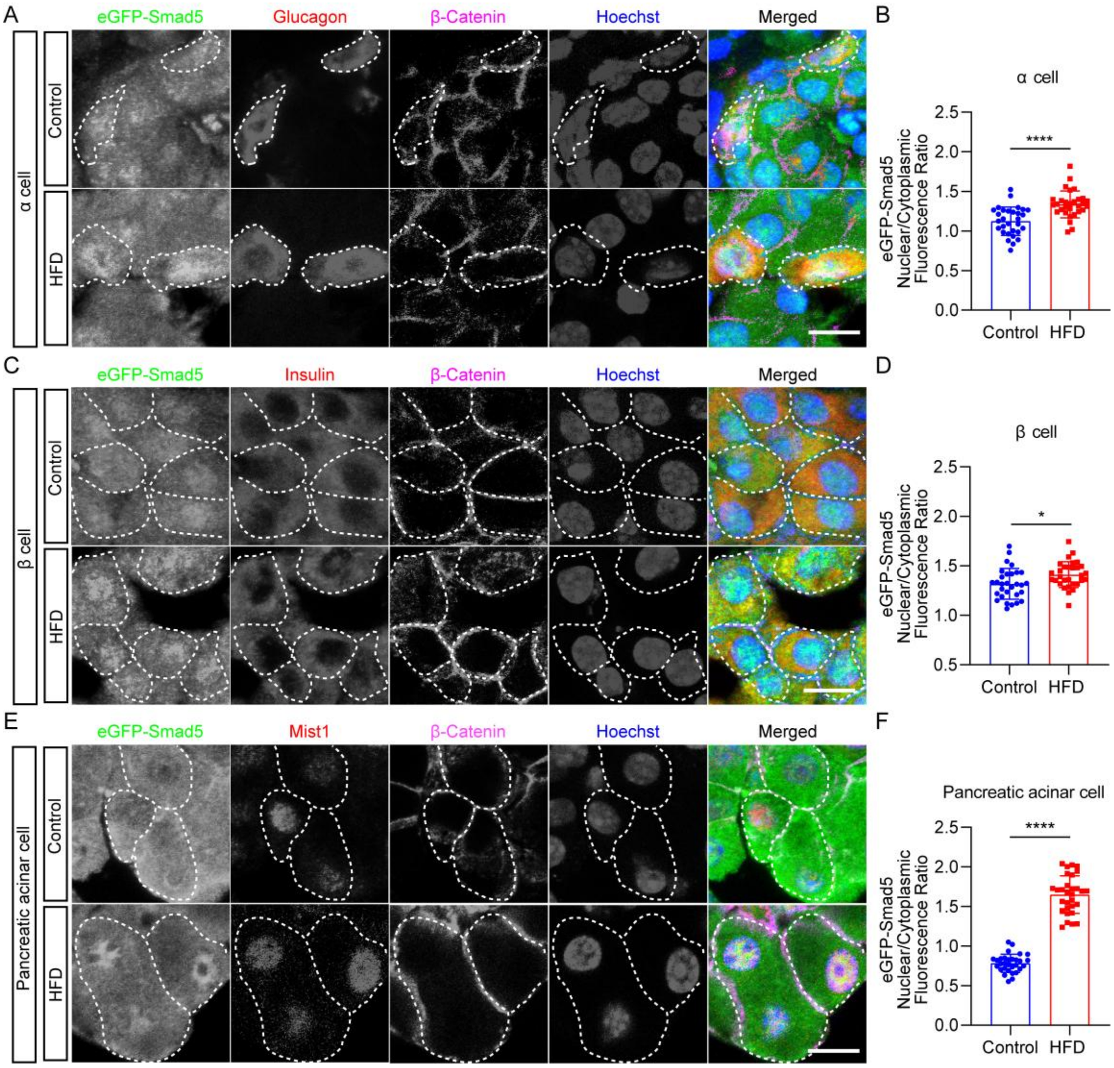

Figure SF15. Aberrant pHc of pancreatic cells in T2DM. Related to **Figure 5.**

A, representative immunofluorescence staining of eGFP-Smad5 (green), Glucagon (red), β-catenin (magenta), and Hoechst 33258 (blue) in α cells in control and T2DM. Scale bar, 10 μm.

B, eGFP-Smad5 nucleocytoplasmic ratio quantification of α cells in control and T2DM in (A). Data are presented as mean ± SD; **** $p < 0.0001$, unpaired two-tailed *t* test; n = 30 cells for both groups.

C, representative immunofluorescence staining of eGFP-Smad5 (green), Insulin (red), β-catenin (magenta), and Hoechst 33258 (blue) in β cells in control and T2DM. Scale

bar, 10 μm.

D, eGFP-Smad5 nucleocytoplasmic ratio quantification of β cells in control and T2DM in (C). Data are presented as mean ± SD; * $p < 0.05$, unpaired two-tailed *t* test; n = 30 cells for both groups.

E, representative immunofluorescence staining of eGFP-Smad5 (green), Mist1 (red), β-catenin (magenta), and Hoechst 33258 (blue) in pancreatic acinar cells in control and T2DM. Scale bar, 10 μm.

F, eGFP-Smad5 nucleocytoplasmic ratio quantification of pancreatic acinar cells in control and T2DM in (E). Data are presented as mean ± SD; **** $p < 0.0001$, unpaired two-tailed *t* test; n = 30 cells for both groups.

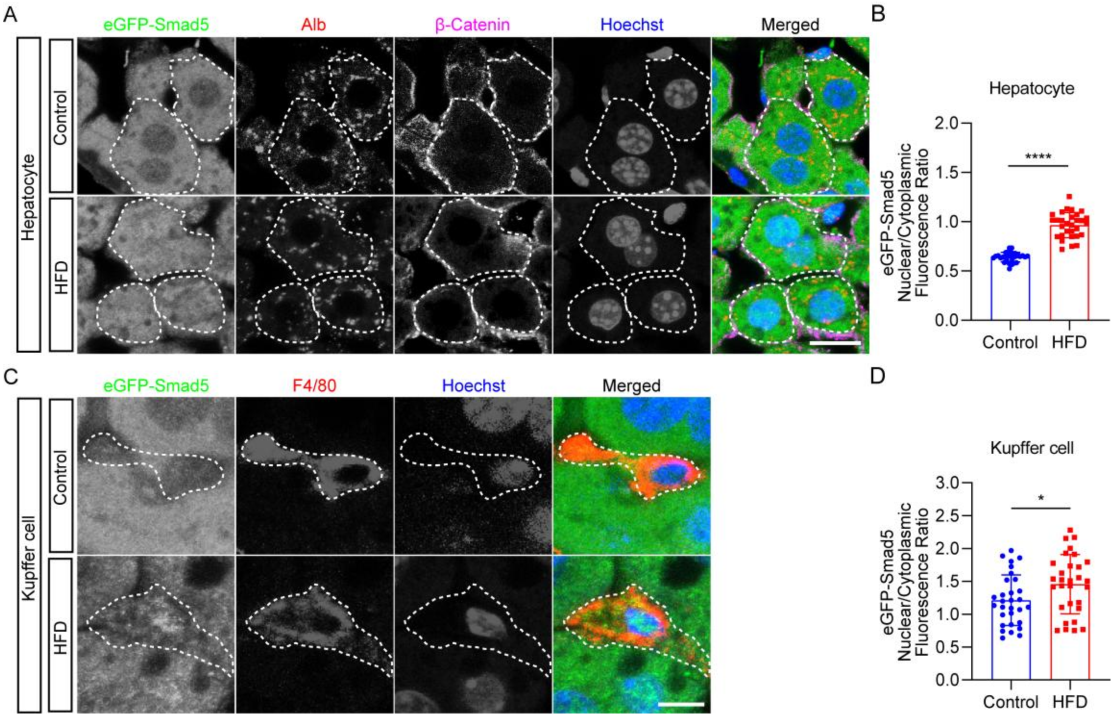

Figure SF16. Aberrant pHc of liver cells in T2DM. Related to **Figure 5.**

A, representative immunofluorescence staining of eGFP-Smad5 (green), Alb (red), β-catenin (magenta), and Hoechst 33258 (blue) in hepatocytes in control and T2DM.

Scale bar, 12.5 μm.

B, eGFP-Smad5 nucleocytoplasmic ratio quantification of hepatocytes in control and T2DM in (A). Data are presented as mean ± SD; **** $p < 0.0001$, unpaired two-tailed *t* test; n = 30 cells for both groups.

C, representative immunofluorescence staining of eGFP-Smad5 (green), F4/80 (red), and Hoechst 33258 (blue) in Kupffer cells in control and T2DM. Scale bar, 4 μm.

D, eGFP-Smad5 nucleocytoplasmic ratio quantification of Kupffer cells in control and T2DM in (C). Data are presented as mean ± SD; * $p < 0.05$, unpaired two-tailed *t* test; n = 30 cells for both groups.

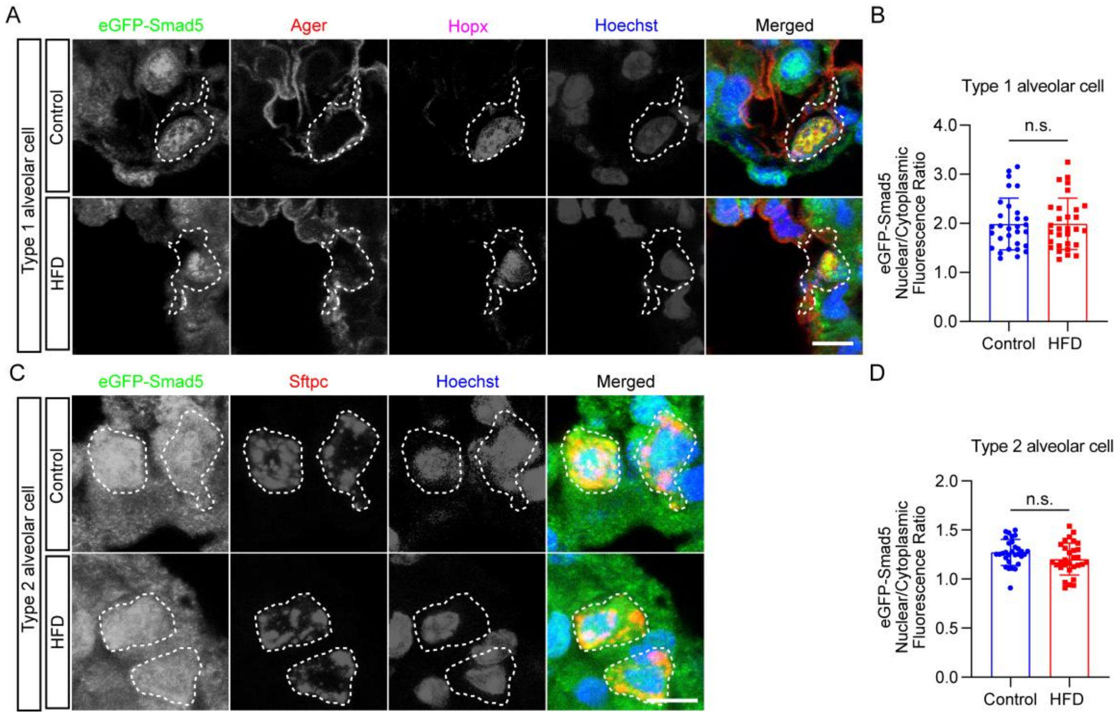

Figure SF17. pHc of lung cells in control and T2DM mice. Related to **Figure 5.**

A, representative immunofluorescence staining of eGFP-Smad5 (green), Ager (red), Hopx (magenta), and Hoechst 33258 (blue) in type 1 alveolar cells in control and T2DM. Scale bar, 8 μm.

B, eGFP-Smad5 nucleocytoplasmic ratio quantification in type 1 alveolar cells in control and T2DM in (A). Data are presented as mean ± SD; n.s., no significant difference, unpaired two-tailed *t* test; n = 30 cells for both groups.

C, representative immunofluorescence staining of eGFP-Smad5 (green), Sftpc (red), and Hoechst 33258 (blue) in type 2 alveolar cells in control and T2DM. Scale bar, 6 μm.

D, eGFP-Smad5 nucleocytoplasmic ratio quantification in type 2 alveolar cells in control and T2DM in (C). Data are presented as mean ± SD; n.s., no significant difference, unpaired two-tailed *t* test; n = 30 cells for both groups.

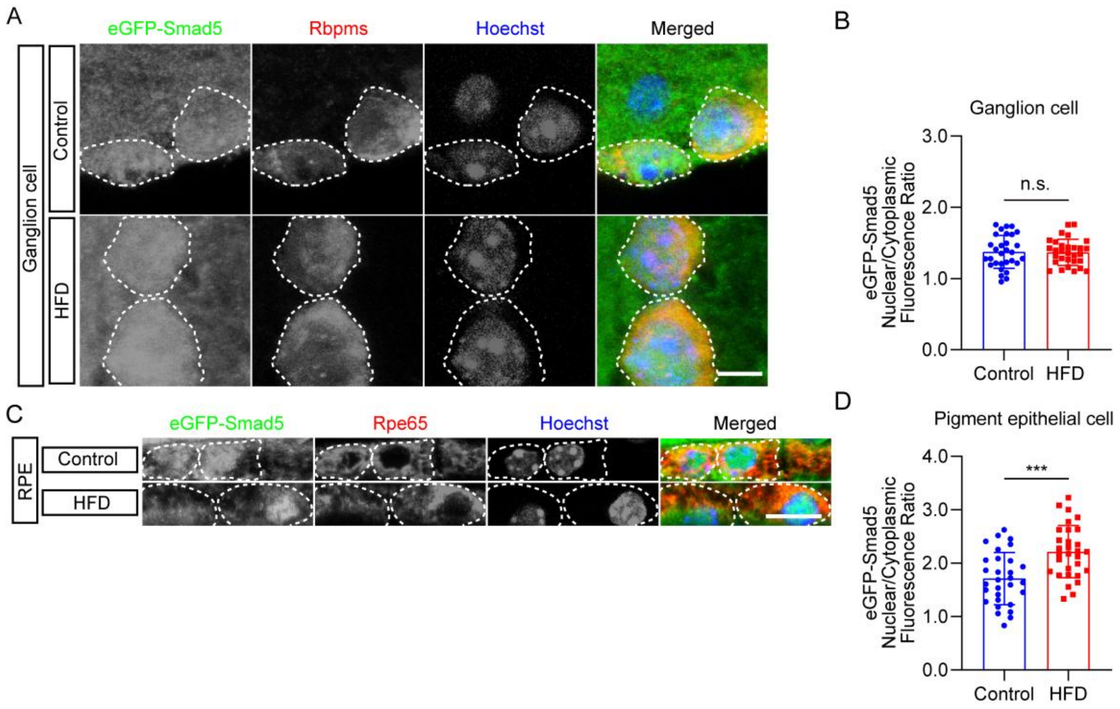

Figure SF18. pHc of retina cells in control and T2DM mice. Related to **Figure 5.**

A, representative immunofluorescence staining of eGFP-Smad5 (green), Rbpms (red), and Hoechst 33258 (blue) in retinal ganglion cells in control and T2DM. Scale bar, 6 μm.

B, eGFP-Smad5 nucleocytoplasmic ratio quantification of retinal ganglion cells in

control and T2DM in (B). Data are presented as mean ± SD; n.s., no significant difference, unpaired two-tailed *t* test; n = 30 cells for both groups.

C, representative immunofluorescence staining of eGFP-Smad5 (green), Rpe65 (red), and Hoechst 33258 (blue) in retinal pigment epithelial cells in control and T2DM. Scale bar, 10 µm.

D, eGFP-Smad5 nucleocytoplasmic ratio quantification of retinal pigment epithelial cells in control and T2DM in (C). Data are presented as mean ± SD; *** $p < 0.001$, unpaired two-tailed *t* test; n = 30 cells for both groups.

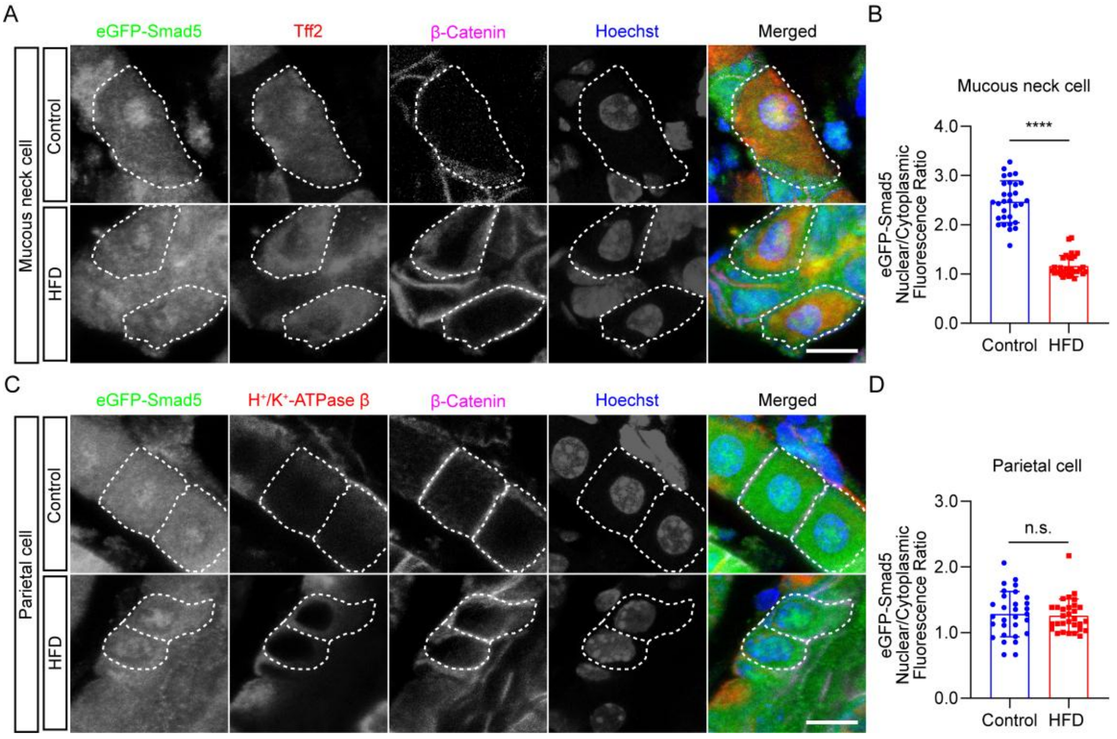

Figure SF19. pHc of stomach cells in control and T2DM mice. Related to **Figure 5.**

A, representative immunofluorescence staining of eGFP-Smad5 (green), Tff2 (red), β-catenin (magenta), and Hoechst 33258 (blue) in mucous neck cells in control and T2DM. Scale bar, 10 µm.

B, eGFP-Smad5 nucleocytoplasmic ratio quantification of mucous neck cells in control

and T2DM in (A). Data are presented as mean ± SD; **** $p < 0.0001$, unpaired two-tailed *t* test; n = 30 cells for both groups.

C, representative immunofluorescence staining of eGFP-Smad5 (green), H^+K^+-ATPase β (red), β-catenin (magenta), and Hoechst 33258 (blue) in parietal cells in control and T2DM. Scale bar, 10 μm.

D, eGFP-Smad5 nucleocytoplasmic ratio quantification of parietal cells in control and T2DM in (C). Data are presented as mean ± SD; n.s., no significant difference, unpaired two-tailed *t* test; n = 30 cells for both groups.

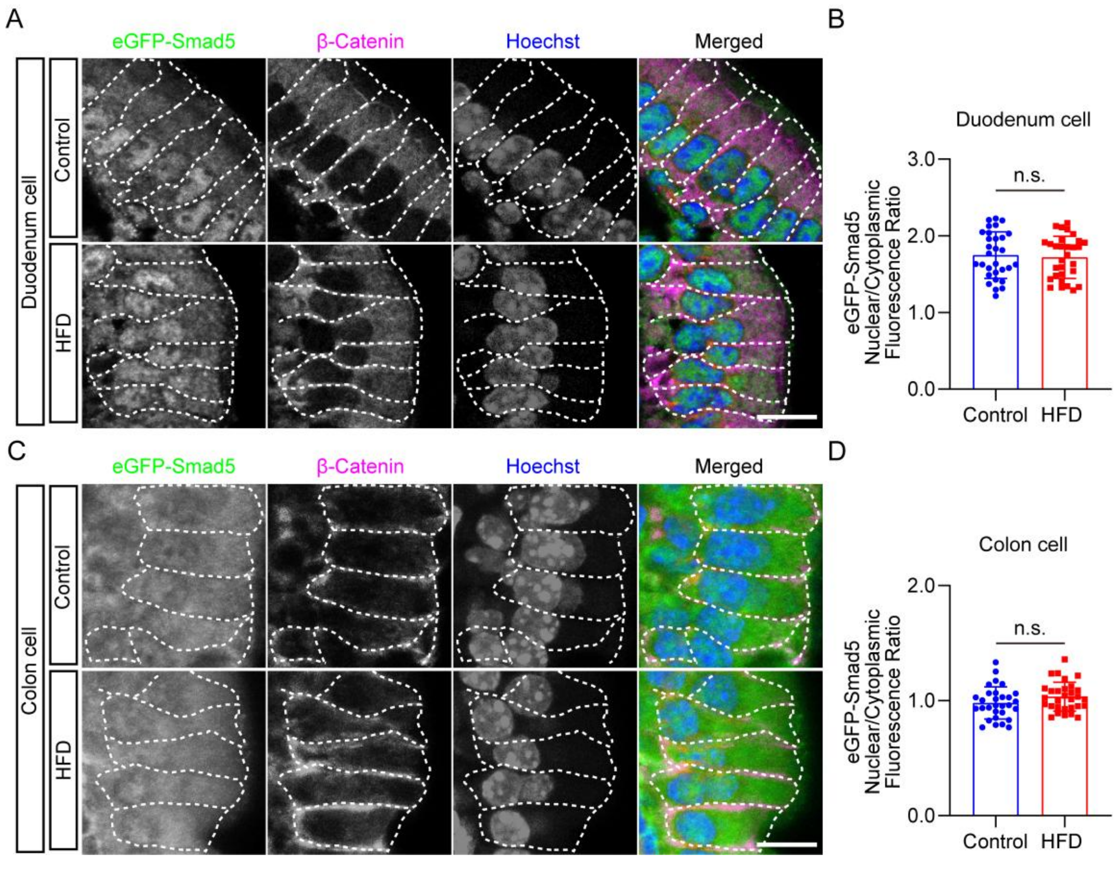

Figure SF20. pHc of gut cells in control and T2DM mice. Related to **Figure 5.**

A, representative immunofluorescence staining of eGFP-Smad5 (green), β-catenin (magenta), and Hoechst 33258 (blue) in duodenum cells in control and T2DM. Scale bar, 10 μm.

B, eGFP-Smad5 nucleocytoplasmic ratio quantification of duodenum cells in control and T2DM in (A). Data are presented as mean ± SD; n.s., no significant difference, unpaired two-tailed *t* test; n = 30 cells for both groups.

C, representative immunofluorescence staining of eGFP-Smad5 (green), β-catenin (magenta), and Hoechst 33258 (blue) in colon cells in control and T2DM. Scale bar, 10 μm.

D, eGFP-Smad5 nucleocytoplasmic ratio quantification of colon cells in control and T2DM in (C). Data are presented as mean ± SD; n.s., no significant difference, unpaired two-tailed *t* test; n = 30 cells for both groups.

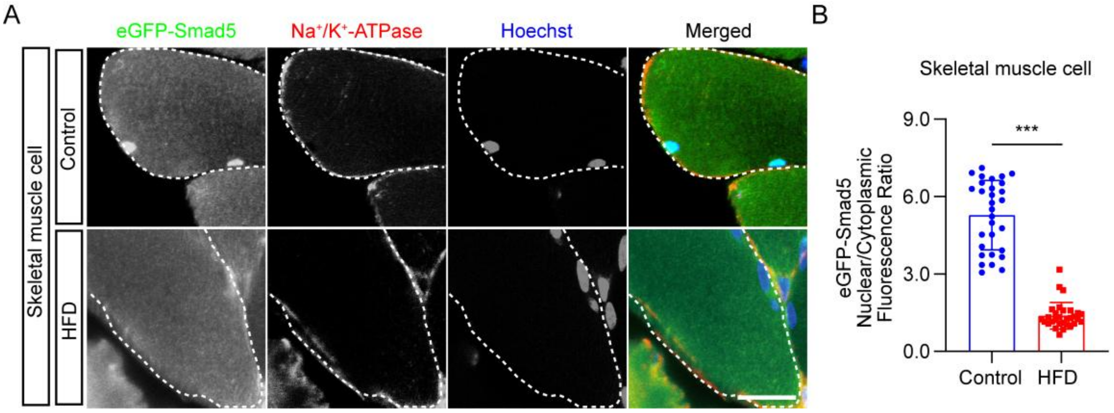

Figure SF21. pHc of skeletal muscle cells in control and T2DM mice. Related to **Figure 5.**

A, representative immunofluorescence staining of eGFP-Smad5 (green), Na^{+}/K^{+}-ATPase (red), and Hoechst 33258 (blue) in skeletal muscle cells in control and T2DM. Scale bar, 20 μm.

B, eGFP-Smad5 nucleocytoplasmic ratio quantification of skeletal muscle cells in control and T2DM in (A). Data are presented as mean ± SD; *** $p < 0.001$, unpaired two-tailed *t* test; n = 30 cells for both groups.

Supporting Information Table 1. The primers used for genotyping.

Prime	Sequence
F1	CTGAACCCCATATCTTCTGTTTC
R1	CACCAGGTTAGCCTTTAAGCCTG
F2	GCTGAGCCAGACCTCCATCG
R2	CAGATGACTACCTATCCTCCC
